# TSTA3 facilitates esophageal squamous cell carcinoma progression through regulating fucosylation of LAMP2 and ERBB2

**DOI:** 10.7150/thno.48225

**Published:** 2020-09-14

**Authors:** Ling Zhang, Yingzhen Gao, Xiaojuan Zhang, Min Guo, Jie Yang, Heyang Cui, Pengzhou Kong, Xia Niu, Yanghui Bi, Jing Xu, Ting Yan, Yanchun Ma, Jian Yang, Yu Qian, Fang Wang, Hongyi Li, Feng Liu, Xiaolong Cheng, Yongping Cui

**Affiliations:** 1 Department of Pathology & Shanxi Key Laboratory of Carcinogenesis and Translational Research of Esophageal Cancer, Shanxi Medical University, Taiyuan, Shanxi 030001, P.R. China.; 2 Department of Oncology (Radiation Oncology), Cancer Institute, Peking University Shenzhen Hospital, Shenzhen Peking University-the Hong Kong University of Science and Technology (PKU-HKUST) Medical Center, Shenzhen 518035, P. R. China.; 3 Department of Gastroenterology, The Second Hospital, Shanxi Medical University, Taiyuan, Shanxi 030001, P.R. China.; 4 Department of Forensic, Shanxi Medical University, Taiyuan, Shanxi 030001, P.R. China.

**Keywords:** ESCC, TSTA3, Fucosylation, LAMP2, ERBB2

## Abstract

**Background:** TSTA3 gene encodes an enzyme responsible for synthesis of GDP-L-fucose as the only donor in fucosylation. This study was designed to explore clinical value, function and underlying mechanism of TSTA3 in the development of esophageal squamous cell carcinoma (ESCC).

**Methods:** Whole genomic sequencing data from 663 ESCC patients and RNA sequencing data from 155 ESCC patients were used to analyze the copy number variation and mRNA expression of TSTA3 respectively. Immunohistochemistry based or not based on the tissue microarrays was used to detect its protein expression. Transwell assay and *in vivo* metastasis assay were used to study the effect of TSTA3 on invasion and metastasis of ESCC. Immunofluorescence was used to analyze fucosylation level. N-glycoproteomics and proteomics analysis, Lens Culinaris Agglutinin (LCA) and Ulex Europaeus Agglutinin I (UEA-I) affinity chromatography, immunoprecipitation, glycosyltransferase activity kit and rescue assay were used to explore the mechanism of TSTA3.

**Results:** TSTA3 was frequently amplified and overexpressed in ESCC. TSTA3 amplification and protein overexpression were significantly associated with malignant progression and poor prognosis of ESCC patients. TSTA3 knockdown significantly suppressed ESCC cells invasion and tumor dissemination by decreasing fucosylation level. Conversely, exogenous overexpression of TSTA3 led to increased invasion and tumor metastasis *in vitro* and *in vivo* by increasing fucosylation level. Moreover, core fucosylated LAMP2 and terminal fucosylated ERBB2 might be mediators of TSTA3-induced pro-invasion in ESCC and had a synergistic effect on the process. Peracetylated 2-F-Fuc, a fucosyltransferase activity inhibitor, reduced TSTA3 expression and fucosylation modification of LAMP2 and ERBB2, thereby inhibiting ESCC cell invasion.

**Conclusion:** Our results indicate that TSTA3 may be a driver of ESCC metastasis through regulating fucosylation of LAMP2 and ERBB2. Fucosylation inhibitor may have prospect to suppress ESCC metastasis by blocking aberrant fucosylation.

## Introduction

Esophageal cancer is the sixth most common cause of cancer death annually worldwide and approximately 70% of global esophageal cancer occurs in China, where the main histologic type is esophageal squamous cell carcinoma (ESCC) 1. Indeed, the majority of ESCC patients reached a relatively locally advanced or even metastatic stage at the first diagnosis 1. Coupled with the lack of early diagnosis markers, prognostic monitoring indicators and therapeutic targets, the five-year survival rate of ESCC patients is only 10% for high grade occurrences 2.

Aberrant glycosylation has been recognized as a hallmark of cancer. Glycosylation is often involved in the folding, polymerization, maturation and transport of protein peptide chains, which plays an important role in the regulation of cellular functions 3, 4. Fucosylation is one of the most common glycosylation modification and many studies revealed that increased fucosylation contributed to many malignant transformation events, such as invasion, metastasis, angiogenesis and immune evasion 5, 6. Abnormal fucosylation has paved a new way for cancer diagnosis, therapy, and prognosis prediction. The most representative is fucosylated alpha-fetoprotein (Fuc-AFP), which is widely used in the early diagnosis of hepatocellular carcinoma because it is more specific than AFP 7, 8. Besides, fucosylated haptoglobin (Fuc-Hpt) is reported to be increased in the sera of patients with different cancers and has been developed as a promising biomarker for the diagnosis of pancreatic cancer 9-12. For therapy, monoclonal antibodies against fucosylated Le Y have been used as potential drugs for immunotherapy of epithelial-derived tumors 13. Inhibition of fucosylation not only suppresses tumor growth but also promotes T cell activation by downregulating PD-1 activity 14. In contrast, the study of fucosylation in esophageal cancer is still lagging. Yu et al. identified that alpha-l-fucosidase was a novel serum biomarker to predict prognosis in early stage ESCC 15. Fucosyltransferase 3, 8 and O-fucosyltransferase 1, 2 genes were identified to be upregulated in esophageal cancer stem-like cells (CSLCs) 16. However, there are few reports of abnormal fucosylation and specific fucosylated proteins in ESCC.

Fucosylation, transferring fucose from GDP-L-fucose to their substrates, is controlled by the actions of fucose synthetase, fucosyltransferase and fucose transporter. GDP-L-fucose, as the only donor of fucosylation, is synthesized by two pathways including *de novo* synthesis and salvage pathways 17. Of which, the *de novo* synthesis pathway synthesizes 90% of GDP-L-fucose 18. GDP-L-Fucose synthase (also called tissue specific transplantation antigen P35B, TSTA3) is one of two key enzymes in this pathway 19. Currently, the study of fucosylation in cancer is mainly focused on the abnormality of fucosyltransferase. The differential expression of the fucosyltransferase has been observed in breast, lung, liver, brain, and thyroid cancer 20. However, fucose synthesis abnormality in cancers has seldom been reported.

In our previous study, we revealed that overexpression of TSTA3 protein was associated with clinical stage and lymph node metastasis in ESCC patients. Moreover, increased TSTA3 expression could independently predict poor prognosis for ESCC patients 21. In this study, we reported the frequent amplification of TSTA3 in ESCC and promoting metastasis of TSTA3 through regulating fucosylation of LAMP2 and ERBB2 in ESCC.

## Results

### Recurrent amplification and upregulation of TSTA3 are associated with patients' metastasis and poor prognosis in ESCC

In our previous study, whole genomic sequencing (WGS) was performed in microneedle-punctured formalin-fixed paraffin-embedded (FFPE) tumor tissues and matched adjacent normal specimens from 508 ESCC patients, in which we reported the largest dataset of genomic profiling of ESCC useful for developing ESCC specific biomarkers for diagnosis and treatment 22. In another parallel unpublished multi-omics study of our group, both WGS and transcriptomic sequencing were performed in FFPE and fresh frozen tumor tissues and matched normal specimens from 155 ESCC patients. Therefore, there were a total of 663 ESCC cases for WGS analysis, of which 155 were also used for RNA analysis.

**Figure 1:**
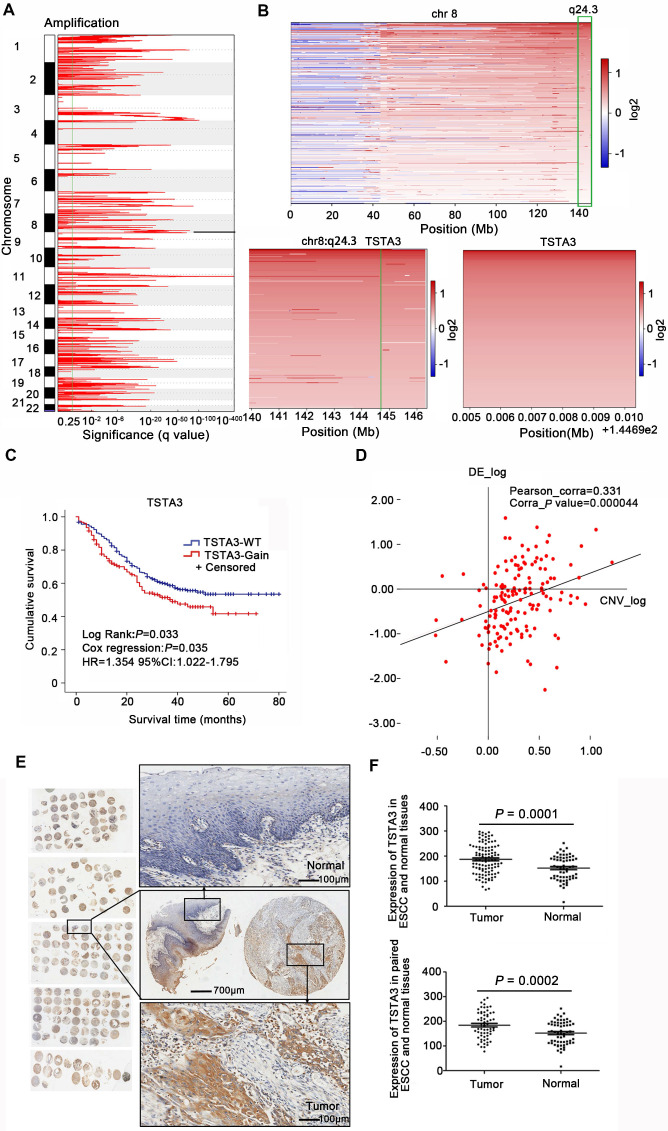
** Copy number amplification and expression of TSTA3 in ESCC. (A)** The significant focal SCNA filtered by GISTC across 663 ESCC genomes. **(B)** Heatmap of CNA log2 ratio of read coverage across 225 ESCC individuals in 8q24.3 covering TSTA3 regions and detected significant amplification of TSTA3. **(C)** Kaplan-Meier's survival analysis between ESCC patients with TSTA3-WT (TSTA3-wild type) and TSTA3-Gain (TSTA3-copy number amplification). The horizontal axis is the survival time and the vertical axis is the percentage of survival. Every cross on the survival curves stands for censored data. Log-rank test *P*-value, Cox regression *P*-value, Hazard Ratio and 95% CI are displayed on the graph.** (D)** Scatter plots of positively correlating TSTA3 amplification and RNA expression. RNA expression values and DNA copy number values are plotted against each other for the 155 cases where WGS and RNA sequencing were performed. **(E)** Representative images of TSTA3 protein expression from FFPE tissue microarrays containing 104 tumors and 60 adjacent normal esophageal tissues by IHC. **(F)** Comparison of the TSTA3 protein expression in non-paired (upper) and paired (bottom) ESCC tumor tissues and normal tissues using *t*-test.

WGS analysis of 663 ESCC cases identified large-scale chromosome amplifications at 1p, 3q, 5p, 8q, 11q, 18p which was consistent with previous findings in ESCC 2, 23 **(Figure 1A)**. Then we focused on somatic focal copy number alterations (FCNA, < 100 kb) using modified GISTIC method showing high-amplitude copy number changes, which have a higher probability of covering cancer genes. This analysis revealed that TSTA3 was present in a significantly amplified focal region around 8q24.3 and TSTA3 locus was amplified in 225 out of 663 tumors (33.9%) **(Figure 1B)**. TSTA3, also known as GDP-fucose synthase (FX), converts cellular GDP-D-mannose into GDP-L-fucose and participates in the *de novo* synthesis pathway of GDP-L-fucose. When the relationship between TSTA3 amplification and clinicopathological characteristics in ESCC cases was analyzed, we observed the following statistically significant patterns: (i) TSTA3 amplification was significantly associated with late clinical stage (*P* = 0.003), (ii) patients harboring TSTA3 amplification exhibited tendency of lymph node metastasis (*P* = 0.002) compared with patients harboring no amplification in TSTA3 **(Table S1)**, and (iii) patients with TSTA3 amplifications had poor survival time than those with wild-type TSTA3 in WGS of 508 FFPE samples [Kaplan-Meier analysis, *P* = 0.033; Cox regression, *P* = 0.035, hazard ratio (HR): 1.354, 95% confidence interval (CI): 1.022-1.795] **(Figure 1C)**. Consistently, the analysis of cancer genome atlas (TCGA) data also revealed that TSTA3 locus was amplified in 58 out of 96 ESCC (60.4%). Moreover, TSTA3 was detected to be amplified in 30.56% of ovarian cancer, 15.69% of breast cancer, 6.67% of head and neck cancer with different amplification frequencies in different cancers **(Figure S1A)**. Furthermore, TSTA3 mRNA was dramatically upregulated in tumor tissues in comparison to that of non-tumor tissues in ovarian cancer, breast cancer, lung cancer, and colon adenocarcinoma from TCGA database **(Figure S1B-C)**.

To investigate the potential role of TSTA3 in ESCC, we first detected the mRNA levels of TSTA3 in transcriptomic sequencing analysis of 155 ESCC cases. As shown in **Figure S2A**, the increase of TSTA3 expression level in tumor samples was not obvious in comparison with non-tumor samples. To investigate the correlation between the mRNA expression of TSTA3 and clinicopathologic features especially the metastasis and survival of ESCC, patients were divided into a high TSTA3 expression group and a low TSTA3 expression group using the ROC curve **(Figure S2B)**. Although the correlation was not statistically significant, TSTA3 mRNA expression levels tended to have a correlation with clinical stage (*P* = 0.067) and lymphatic metastasis (*P* = 0.087) **(Table S2)**. Kaplan-Meier analysis showed the patients in the TSTA3-high group had a shorter survival time than those in the TSTA3-low group (*P* = 0.043)** (Figure S2C)**. Moreover, integration analysis of the WGS and transcriptome was used to determine whether mRNA expression of TSTA3 was associated with copy number amplification. The results showed that there was a positive correlation between expression levels of TSTA3 and estimates of copy numbers to some extent (Pearson correlation coefficient = 0.331; *P*-value = 0.000) **(Figure 1D)**, consistent with the analysis of ESCC tissues without paired normal tissue in TCGA database and the analysis of 27 ESCC cell lines in Cancer Cell Line Encyclopedia (CCLE) **(Figure S2D-E)**. These results indicated the upregulation of TSTA3 expression might be caused by copy number gain in ESCC.

The pattern of TSTA3 amplification and mRNA expression in ESCC promoted us to further investigate the expression of TSTA3 protein and the association with metastasis and prognosis of ESCC patients. TSTA3 protein levels were measured in 104 primary ESCC tissues and 60 paired adjacent normal tissues on tissue microarray. The immunohistochemistry results showed that TSTA3 was mainly expressed in the cytoplasm of ESCC tissues** (Figure 1E)**. The protein expression level of TSTA3 in 104 ESCC tissues was significantly higher than that in 60 adjacent normal esophageal squamous epithelium. The difference was also significant in 60 paired cancer tissues and adjacent normal tissues (*P* = 0.000) **(Figure 1F)**. Correlation analysis between protein expression and clinicopathological factors showed that the high expression of TSTA3 was also significantly associated with late clinical stage, more lymph node metastasis and poor prognosis for ESCC patients 21. Combining with TSTA3 as a fucose synthesis related enzyme, we speculated that abnormal fucosylation might participate in the progress of ESCC and TSTA3 might serve as a novel biomarker for prognosis of ESCC patients.

### TSTA3 promotes cell migration and invasion in ESCC

**Figure 2:**
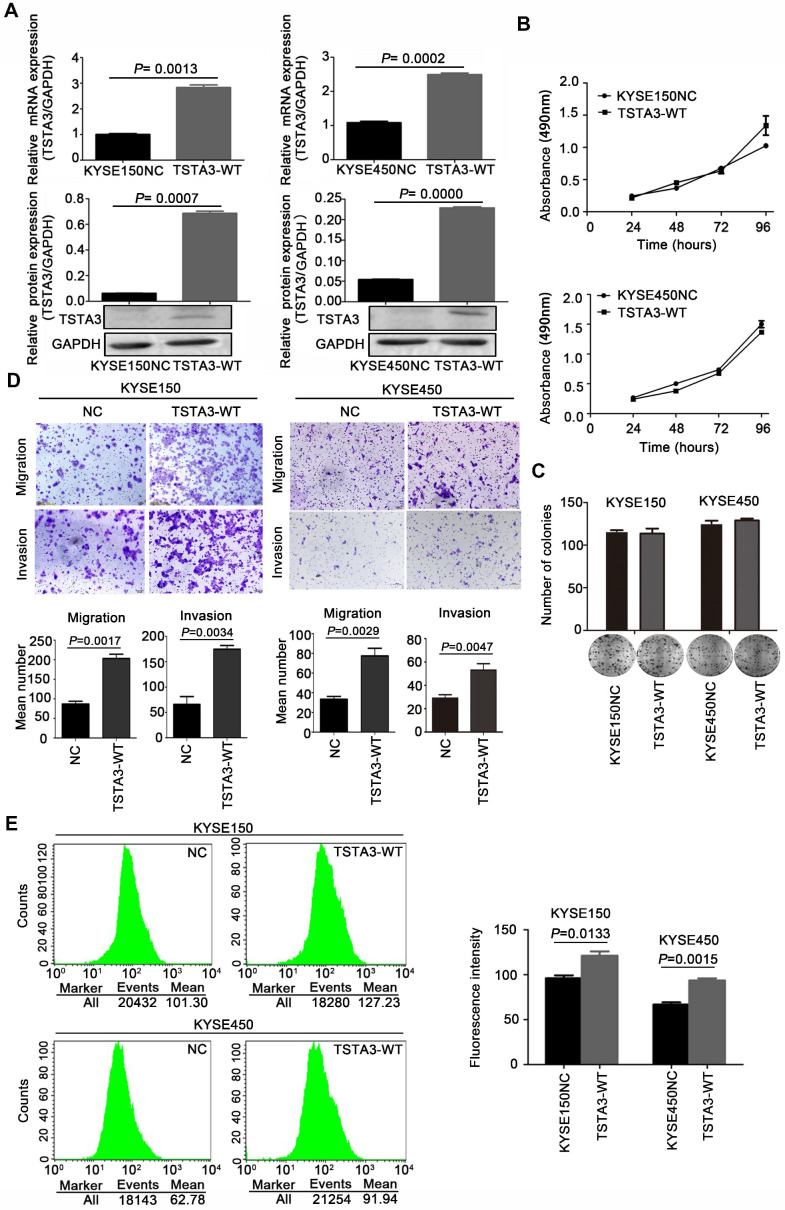
** TSTA3 overexpression promoted ESCC cells migration and invasion *in vitro*. (A)** The overexpression efficiency of TSTA3 in KYSE150 (left) and KYSE450 (right) cells were confirmed by qPCR (upper) and western blot (bottom).** (B)** Cell proliferation ability of KYSE150 (upper) and KYSE450 (bottom) cells stably overexpressing TSTA3 (TSTA3-WT group) or control vector (NC group) was analyzed by MTT. **(C)** The ability of colony formation was analyzed by colony formation assay. **(D)** Transwell migration and invasion assays on KYSE150 and KYSE 450 cells overexpressing TSTA3 or control vector. TSTA3 overexpression promoted cell migration and invasion of KYSE150 (left) and KYSE450 (right) cells. Bottom is a bar chart for quantitative analysis. **(E)** LCA fluorescence of KYSE150 (upper) and KYSE450 (bottom) cells overexpressing TSTA3 or control vector were analyzed by FACS. Right panel shows quantitation of fluorescence. All data are presented as the mean ± standard deviation and three independent experiments.

To clarify biological functions of TSTA3 in ESCC, we firstly measured endogenous expression levels of TSTA3 in several ESCC cell lines by RT-qPCR. The different expression level in different ESCC cell lines was showed in **Figure S3**. Among them, KYSE150 and KYSE450 ESCC cells with low endogenous expression were used for transfecting exogenous wild-type TSTA3. The efficiency of overexpression was confirmed by RT-qPCR and western blot **(Figure 2A).** Although overexpression of wild-type TSTA3 had no effect on the growth and colony formation of ESCC **(Figure 2B-C)**, TSTA3 exogenous overexpression markedly promoted cell invasion and migration of KYSE150 and KYSE450 **(Figure 2D)**. As expected, overexpression of wide-type TSTA3 also resulted in increased core fucosylation and terminal fucosylation identified by Lens Culinaris Agglutinin (LCA) and Ulex Europaeus Agglutinin I (UEA-I) lectin respectively** (Figure 2E and Figure S4A-B)**.

**Figure 3:**
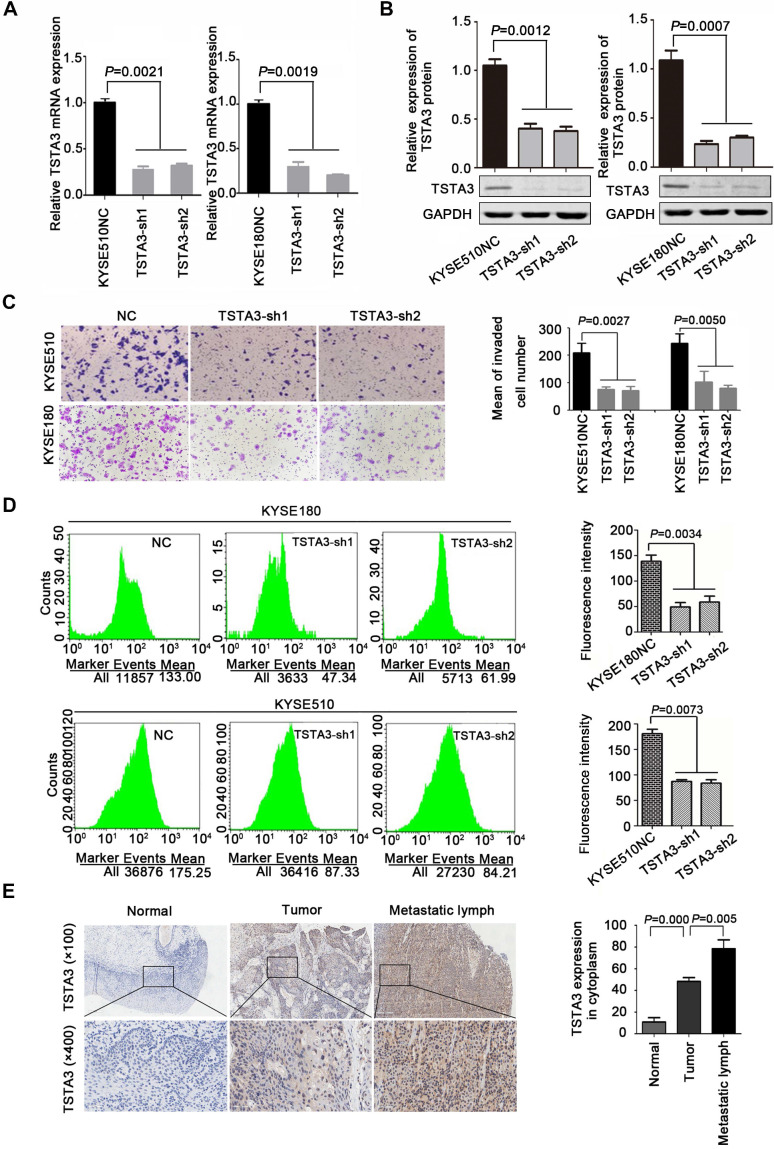
** TSTA3 silencing reduced ESCC cells invasion and fucosylation level. (A)** Relative TSTA3 mRNA levels in KYSE510 (left) and KYSE180 (right) cells treated with the lentivirus expressing TSTA3-shRNA (sh1 or sh2) or sh-NC (negative control) were assessed by qPCR.** (B)** Relative expression of TSTA3 protein in KYSE510 (left) and KYSE180 (right) cells treated with the lentivirus expressing TSTA3-shRNA (sh1 or sh2) or sh-NC were assessed by western blot. **(C)** Transwell invasion assays on KYSE510 (upper) and KYSE180 (bottom) with TSTA3 knockdown and control group. Right is a bar chart for quantitative analysis.** (D)** UEA-I fluorescence of KYSE180 (upper) and KYSE510 (bottom) cells was analyzed by FACS. The right panel shows quantitation of UEA-I fluorescence. All data are presented as the mean ± standard deviation and three independent experiments. **(E)** Representative immunohistochemistry images of TSTA3 expression in tumor tissues, matched normal tissues and metastatic lymph node from paraffin-embedded formalin-fixed 11 ESCC tissues. Right: TSTA3 expression in metastatic lymph node tissue showed significantly higher expression than that of matched tumor and normal tissues.

Additionally, further functional experiments of TSTA3 knockdown were also performed in KYSE180 and KYSE510 cells with high endogenous TSTA3 level in which effective knockdown efficiency has been validated by RT-qPCR and western blot **(Figure 3A-B)**. As expected, TSTA3 knockdown had no effect on cell proliferation **(Figure S5A-B)** but attenuated the invasion ability of both KYSE180 and KYSE510 cells** (Figure 3C)**. Moreover, knockdown of TSTA3 also decreased fucosylation level identified by immunofluorescence of UEA-I lectin in KYSE510 and KYSE180 ESCC cells** (Figure 3D).** These results indicated that TSTA3 might promote metastasis of ESCC by abnormal fucosylation modification.

In order to further determine the role of TSTA3 in ESCC metastasis, we detected TSTA3 protein level using immunochemistry in 28 metastatic lymph node tissues from eleven ESCC cases. Both tumor tissues and paired normal tissues of these eleven patients were also available. Details of clinicopathological features were summarized in **Table S3**. We observed that TSTA3 was strongly stained in the cytoplasm of metastatic tumor tissues. Importantly, the TSTA3 expression in metastatic lymph node tissue showed significantly higher than that of matched tumor and normal tissues** (Figure 3E)**.

**Figure 4:**
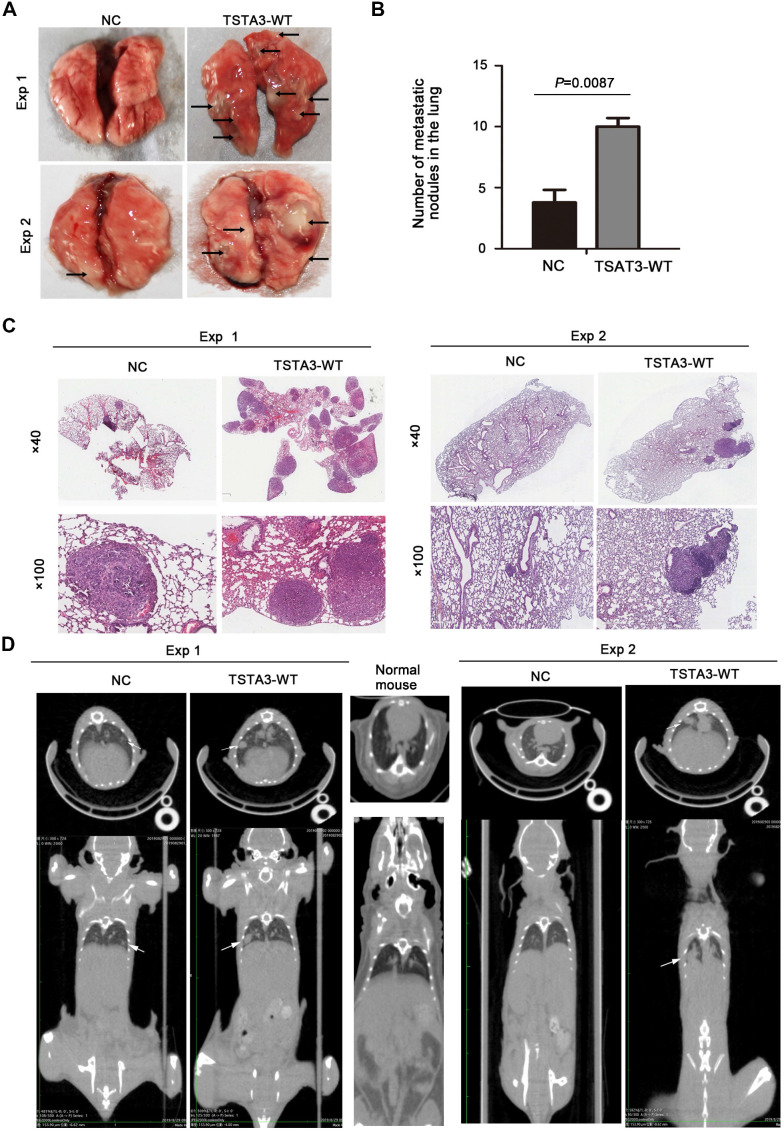
** TSTA3 overexpression increased *in vivo* ESCC metastasis. (A)** Representative images of metastatic tumor nodules in the lung of nude mice intravenously injected with KYSE150 cells stably overexpressing TSTA3 (TSTA3-WT) or control vector (NC). **(B)** Number of metastatic tumor nodules in the lung were compared between nude mice injected with TSTA3-WT and control cells and statistically analyzed. All data are presented as the mean ± standard deviation. **(C)** Representative images of H&E staining in metastatic tumor nodules in the lung section of nude mice. **(D)** Representative images transverse (upper) and coronal (bottom) sections of CT images of nude mice. White arrows show water-density nodule lung in CT.

To further confirm the role of TSTA3 in promoting metastasis in ESCC, we injected KYSE150 cells with empty vector (NC group) and stably overexpressing TSTA3 (TSTA3-WT group) into the tail vein of nude mice and observed pulmonary and liver metastasis. Compared with the NC group, the TSTA3-WT group had worse physical status. Although no obvious and visible metastatic nodules were observed in the liver, the mice injected into ESCC cells with TSTA3-WT exhibited significantly increased lung metastatic nodules compared with the control mice injected into ESCC cells with vector control, which was confirmed by the quantitative analysis and HE staining (*P* = 0.009) **(Figure 4A-C)**. Moreover, small-animal ^18^F-FDG PET/CT was also used to observe pulmonary metastases of two groups. Although the PET images showed no high uptake of ^18^F-FDG in the metastatic ESCC areas, the CT images exhibited round and obvious metastatic nodules in the lung parenchyma and near the visceral pleura in TSTA3-WT group compared with NC group **(Figure 4D)**. Further immunohistochemical analysis confirmed the overexpression of TSTA3 in metastatic tumor tissue of nude mice **(Figure S6).** Our findings provided compelling evidence that wild-type TSTA3 overexpression promoted the ability of ESCC cells to colonize distal organs.

### N-glycoproteomics reveals glycoproteins and signaling pathways that may be associated with TSTA3-mediated ESCC metastasis

**Figure 5:**
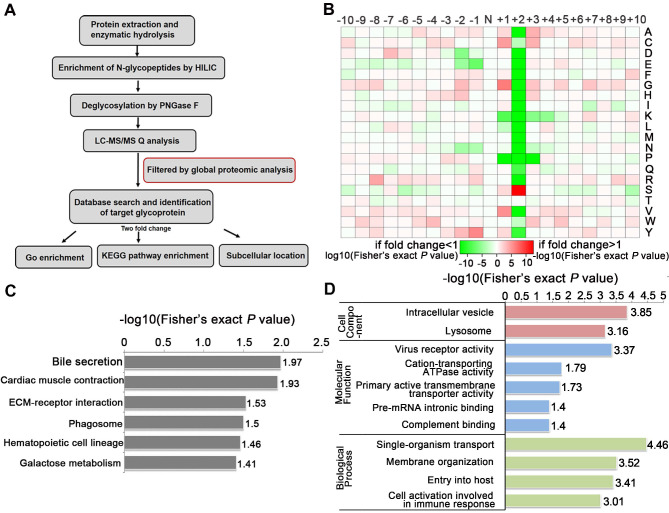
** N-glycoproteomics analysis identified glycoproteins mediating the effects of TSTA3 on ESCC metastasis**. **(A)** Schematic illustration of our systems biology approach to identify N-glycoproteins mediating the effects of TSTA3 on ESCC metastasis. **(B)** Motif analysis of glycosylation sites identified in N-glycoproteomics analysis. **(C)** KEGG pathways analysis of the differentially expressed glycoproteins in KYSE150 NC group and TSTA3-WT group. **(D)** The classification of differentially expressed glycoproteins in cell components, molecular function and biological process (N = 3 biological triplicates).

To identify the glycosylated proteins that mediate the effects of TSTA3 on ESCC metastasis, we applied LC-MS/MS technology featuring proteomics and N-glycoproteomics to compare the expression level of proteins and glycoproteins in negative control and TSTA3 overexpressed KYSE150 cells. Because seemingly differential expressed glycoproteins may be attributed to changes in protein expression, to ensure high confidence, the standard of localization probability > 0.75 was used to filter the data and the quantified values of the filtered glycosylation modification sites were normalized by protein quantification. Global proteomics and N-glycoproteomics data generation quality control indicated that proteomics and N-glycoproteomics analysis system was robust **(Figure S7)**. Schematic illustration of our systems biology was shown in **Figure 5A**. A total of 1100 N-glycosylation sites in 575 glycoproteins were identified, of which 642 sites of 371 proteins contained quantitative information. Among these glycoproteins, we found that 37 glycoproteins were up-regulated and 87 were down-regulated with fold change over 2.0. All the detailed data of differentially expressed glycoproteins and glycosylation sites were listed in **Table S4**. Also as shown in **Figure 5B**, N-glycosylation sequence motif analysis revealed that the most significantly enriched motif was the classical N-glycosylation sequence: N-X-S. Notably, serine occurred more frequently than threonine at the second position. The differentially expressed glycoproteins were implicated in phagosome process, ECM-receptor interaction, galactose metabolism (e.g., GLB1, GAA) and so on (*P* < 0.05)** (Figure 5C)**. For phagosome, lysosome-associated membrane glycoprotein (e.g., LAMP1, LAMP2), TFRC and so on were identified to be differentially expressed glycoproteins. For ECM-receptor interaction, integrin (e.g., ITGB5, ITGA3, ITGB1) and several extracellular matrix (e.g., LAMC1, LAMA5) were identified to be differentially expressed glycoproteins. The above KEGG-enrichment results suggested that the interaction with the extracellular matrix regulated by glycosylation and N- glycosylation modification within lysosome luminal domain were central and typical characteristics during TSTA3 mediated cancer progression. It has been reported that lysosomes tended to shift from perinuclear to plasma membrane for release of contents facilitating migration/invasion of cancer cells 24 and glycosylation of integrin was well-recognized to modulate metastasis of cancer cell through changing location and cell communication 25. The detailed mechanism of differential glycoprotein-involved signaling in TSTA3-mediated ESCC metastasis remains to be further studied. Consistently, the cellular component analysis revealed that the differentially expressed glycoproteins mainly distributed in intracellular vesicle and lysosome. These glycoproteins were mainly associated with cation-transporting ATPase activity, primary active transmembrane transporter activity in molecular function analysis **(Figure 5D)**.

### LAMP2 and ERBB2 are core fucosylated and terminal fucosylated target modified protein of TSTA3

**Figure 6:**
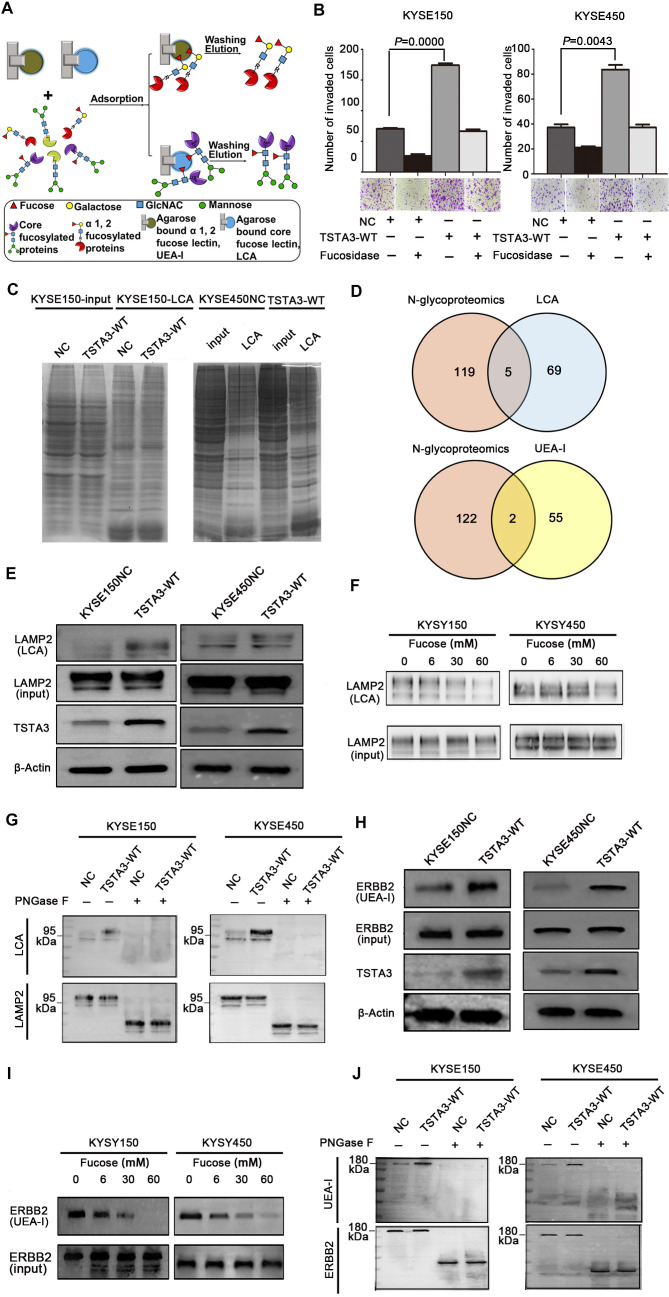
** Identification of fucosylated glycoproteins in ESCC revealed regulators of invasion and metastasis. (A)** Schematic illustration of the experimental approach showing affinity enrichment of core-fucosylated and α-1,2-fucosylated proteins by LCA and UEA-I lectin affinity chromatography respectively. **(B)** The effect of enriched fucosylated protein by UEA-I lectin with or without α-1,2-fucosidase on KYSE150 and KYSE450 cell invasion. **(C)** Coomassie brilliant blue (CBB) staining of gels of whole cell lysate proteins and LCA enriched fucosylated proteins in TSTA3-WT and control group. **(D)** Number of overlapping proteins between lectin enriched proteins identified by in gel mass spectrometry analysis and differentially expressed glycoproteins in N-glycoproteomics data.** (E)** LCA affinity chromatography of whole-cell lysates of ESCC cells transfected with TSTA3-WT and NC followed by western blot with LAMP2 antibody. **(F)** A representative western blot of LCA-affinity purified LAMP2. LCA-affinity purification was done in the absence or presence of various concentrations of α-L-fucose (0, 6, 30 and 60 mM). **(G)** LAMP2 immunoprecipitation from whole-cell lysates of KYSE150 and KYSE450 cells transfected with TSTA3-WT and NC. Anti-LAMP2 immunoprecipitate was treated with or without PNGase F and blotted with biotinylated LCA or LAMP2 antibody. **(H)** UEA-I affinity chromatography of whole-cell lysate of ESCC cells transfected with TSTA3-WT and NC followed by western blot with ERBB2 antibody.** (I)** A representative western blot of UEA-I-affinity purified ERBB2. UEA-I-affinity purification was done in the absence or presence of various concentrations of α-L-fucose.** (J)** ERBB2 immunoprecipitation from whole-cell lysates of KYSE150 and KYSE450 cells transfected with TSTA3-WT and NC. Anti-ERBB2 immunoprecipitate was treated with or without PNGase F and blotted with biotinylated UEA-I or ERBB2 antibody.

Next, we extracted total protein of negative control and TSTA3 overexpressed ESCC cells and performed affinity enrichment for core-fucosylated proteins and α-1,2 fucosylated proteins using LCA and UEA-I lectin chromatography respectively **(Figure 6A)**. After elution and lyophilization, the enriched total fucosylated protein was dissolved and added to the upper compartment of the transwell chamber together with ESCC cells, the results showed that fucosylated protein enriched by UEA-I lectin in TSTA3-WT group promoted cell invasion more strongly than that of NC group. Interestingly, this difference decreased dramatically after fucosylated proteins were treated with α-L-fucosidase **(Figure 6B)**. To further identify fucosylated proteins mediating the promotion effects of TSTA3 in ESCC metastasis, lectin enrichment blot and LC/MS/MS analysis of whole in-gel digestion were used to identify the differential proteins in TSTA3-WT and NC group **(Figure 6C)** (UEA-I lectin enrichment blot was directly used for whole in-gel digestion and LC/MS/MS analysis). We integrated normalized N-glycoproteomics data with in-gel mass spectrometric analysis, in which we focused on the fucosylated proteins identified only in TSTA3 overexpressed ESCC cells but not in control cells. Five and two candidate proteins were identified in the LCA enrichment and UEA-I enrichment respectively **(Figure 6D)**. Proteins identified by LCA lectin involved in metastasis included lysosomal associated membrane protein 2 (LAMP2), IKBKB interacting protein (IKBIP), lamin A/C (LMNA), CD276 and activated leukocyte cell adhesion molecule (ALCAM). Proteins identified by UEA-I lectin included solute carrier family 39 member 14 (SLC39A14) and glucosidase alpha, acid (GAA). To validate our proteomic analysis, we further examined the fucosylation state of these proteins. LCA lectin affinity enrichment followed by western blot showed increased fucosylated LAMP2 levels in TSTA3-WT compared with NC transfected KYSE150 and KYSE450 cells **(Figure 6E)**, consistent with higher core fucosylation on the protein, while input showed no differences in the expression level of total protein. Furthermore, with the increase of α-L-fucose concentration in affinity reaction system, the amount of LAMP2 bound by LCA decreased correspondingly **(Figure 6F)**. Consistently, immunoprecipitation (IP) of LAMP2 followed by LCA blot showed increased LCA binding to LAMP2 proteins in TSTA3-WT compared with NC transfected KYSE150 and KYSE450 cells **(Figure 6G)**. The observed differences disappeared upon PNGase treatment, which removes all forms of N-linked glycosylation. Western blots confirmed equal amounts of IP input in each condition **(Figure 6G)**.

For UEA-I affinity-enriched proteins, SLC39A14A has not been reported to be associated with tumor metastasis and GAA itself is related to glucose metabolism. We were more interested in fucosylation of erb-b2 receptor tyrosine kinase 2 (ERBB2) which was identified in mass spectrometric analysis and fucosylation of ERBB2 has not been reported in ESCC. Intriguingly, UEA-I lectin enrichment followed by western blot showed significantly increased fucosylated ERBB2 levels in TSTA3-WT compared with NC transfected KYSE150 and KYSE450 cells **(Figure 6H)**, while input showed no differences in the expression level of total ERBB2 protein. Furthermore, with the increase of α-L-fucose concentration and competitive binding to lectin, the amount of ERBB2 enriched by UEA-I also decreased correspondingly **(Figure 6I)**. Consistently, immunoprecipitation (IP) of ERBB2 followed by UEA-I blot showed increased UEA-I binding to ERBB2 proteins in TSTA3-WT group compared with NC group. The observed differences also disappeared upon PNGase treatment, despite equal amounts of IP input of each condition **(Figure 6J)**. The densitometries on the western blots of **Figure 6E-J** were performed and exhibited in the **Figure S8**. These results demonstrated that LAMP2 and ERBB2 were probably core fucosylated and terminal fucosylated mediators of the pro-invasive effects of TSTA3.

### LAMP2 and ERBB2 are mediators of TSTA3-induced pro-invasive effects in ESCC

**Figure 7:**
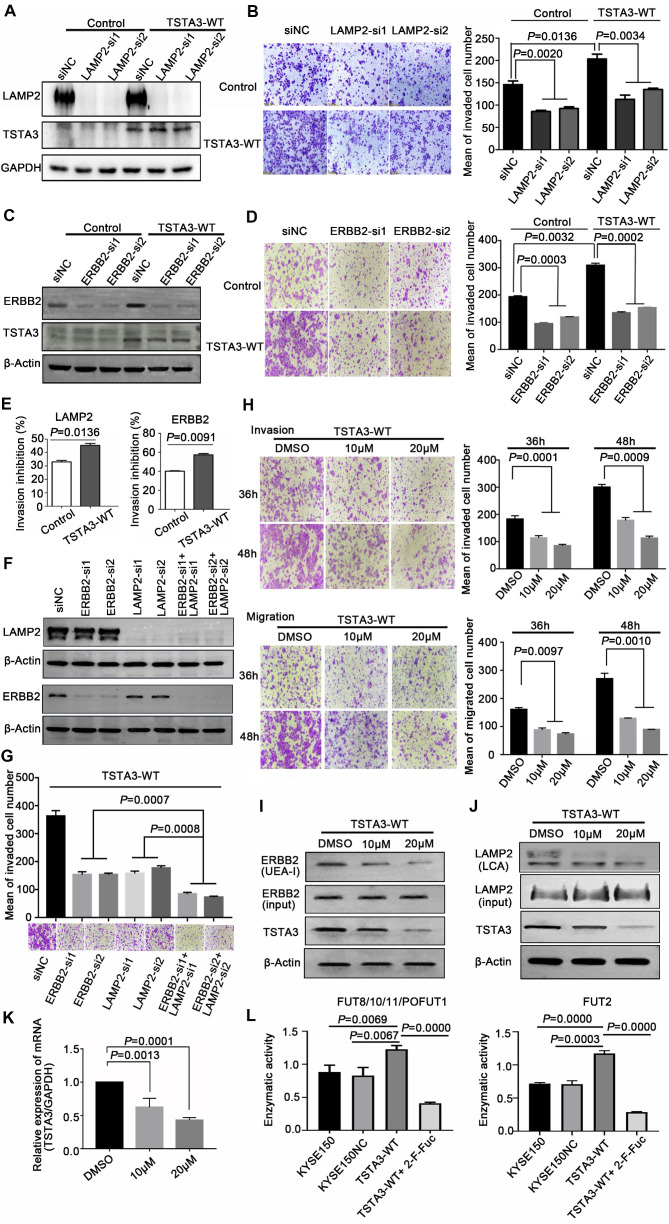
** LAMP2 and ERBB2 were mediators of the pro-invasive effects of TSTA3. (A)** Knockdown efficiency of LAMP2 was detected by western blot. KYSE150 cells stably overexpressing TSTA3 or control vector were transfected with scramble control or LAMP2 siRNA (si1 and si2). **(B)** Transwell invasion assay on KYSE150 cells stably overexpressing TSTA3 or control vector and transfected with scramble control or LAMP2 siRNA (si1 and si2). **(C)** Knockdown efficiency of ERBB2 was detected by western blot. KYSE150 cells stably overexpressing TSTA3 or control vector were transfected with scramble control or ERBB2 siRNA (si1 and si2).** (D)** Transwell invasion assay on KYSE150 cells stably overexpressing TSTA3 or control vector and transfected with scramble control or ERBB2 siRNA (si1 and si2). N = 5 fields per replicate; 3 replicates per condition. **(E)** Invasion inhibition rates of LAMP2 and ERBB2 knockdown in TSTA3 overexpressing KYSE150 cells and control cells. **(F)** Knockdown efficiency detection of LAMP2 and ERBB2 in TSTA3 overexpressing KYSE150 cell lines. **(G)** Synthetic effect of invasion inhibition in TSTA3 overexpressing KYSE150 cells with both LAMP2 and ERBB2 knockdown. **(H)** Transwell chambers assay showed inhibition of invasion and migration by peracetylated 2-F-Fuc treatment in KYSE150 cells stably overexpressing TSTA3. All Data are mean ± standard deviation; each experiment was performed in triplicate.** (I)** UEA-I affinity chromatography followed by western blot with ERBB2 antibody after 2F-Fuc treatment.** (J)** LCA affinity chromatography followed by western blot with LAMP2 antibody after 2F-Fuc treatment. **(K)** 2-F-Fuc treatment decreased the mRNA expression of TSTA3.** (L)** Altered fucosyltransferases activity upon TSTA3 overexpression and 2-F-Fuc treatment in KYSE150 cells.

LAMP2 (CD107b) is a highly glycosylated protein which is normally present in the lysosomal membranes and occasionally in cell membranes, known to regulate cell invasion and migration in several cancers 26. To further validate that LAMP2 may be a mediator of the pro-invasive effects of TSTA3, we knocked down LAMP2 in stably TSTA3 overexpressing KYSE150 cells and the knockdown efficiency was validated by western blot **(Figure 7A)**. Subsequent invasion experiments showed that silencing of LAMP2 strongly counteracted the effects of pro-invasion of TSTA3 overexpression. Moreover, LAMP2 knockdown in parental cell also significantly inhibited cell invasion **(Figure 7B)**. Overall, our data indicated that core-fucosylated LAMP2 served as TSTA3-downstream effectors.

As a member of the epidermal growth factor (EGF) receptor family, ERBB2 overexpression and activation in cancer metastasis have been well recognized. However, the role of fucosylation of ERBB2 in cancer metastasis, including ESCC, is largely unknown. In keeping with the known impact of ERBB2 on cell invasion, silencing of ERBB2 triggered a significant decrease *in vitro* cell invasion. To further validate that ERBB2 is a mediator of the pro-invasive effects of TSTA3, we knocked down ERBB2 in stably TSTA3 overexpressing KYSE150 cells and the knockdown efficiency was validated by western blot **(Figure 7C)**. Subsequent invasion experiments showed that silencing of ERBB2 also strongly counteracted the effects of pro-invasion of TSTA3 overexpression, supporting α-1,2-fucosylated ERBB2 as a key mediator of TSTA3 pro-metastatic effects in ESCC **(Figure 7D)**. Actually, for both LAMP2 and ERBB2 knockdown, the inhibitory effect of invasion in TSTA3 overexpressing ESCC cells was more significant than that of the blank KYSE150 control cells **(Figure 7E)**. Not only that, our further study demonstrated that there was a synergistic effect of inhibiting invasion if both LAMP2 and ERBB2 were down-regulated in TSTA3 overexpressed KYSE150 cells **(Figure 7F-G).** Down-regulation of LAMP2 or ERBB2 alone may affect cell growth, proliferation, and cell death in ESCC (**Figure S9**).

Finally, we tested the potential therapeutic application of fucosylation inhibitor in ESCC. We treated TSTA3 overexpressed KYSE150 cells with peracetylated 2-fluoro 2-deoxy-L-fucose (peracetylated 2-F-Fuc) which is a fucose analog with a fluorine group, to examine migration and invasion ability of the cells. As expected, peracetylated 2-F-Fuc treatment resulted in profound inhibition of cell migration and invasion at the concentration of 10 μM and 20μM **(Figure 7H)**. After peracetylated 2-F-Fuc treatment, UEA-I and LCA lectin enrichment followed by western blot showed significant decrease of fucosylated ERBB2 and LAMP2 levels despite inputs showed no differences in the expression level of total protein. Meanwhile, peracetylated 2-F-Fuc also reduced the expression of TSTA3 protein to some extent **(Figure 7I-J)**. Further RT-qPCR analysis found the decreased mRNA expression of TSTA3 after peracetylated 2-F-Fuc treatment **(Figure 7K)**. Therefore, accumulation of GDP-2F-Fuc through salvage pathways from 2F-Fuc possibly reduced the expression of TSTA3 at transcriptional level and shut down the *de novo* synthesis of GDP-Fucose via a feedback loop. Meanwhile, altered enzymatic activity of fucosyltransferases impacted by TSTA3 overexpression and peracetylated 2-F-Fuc treatment may be involved in altered fucosylation of key proteins mediating tumor invasion **(Figure 7L)**. The results suggested that the fucose analog could be used to suppress cancer metastasis by blocking aberrant fucosylation in ESCC.

## Discussion

TSTA3, also known as GDP-fucose synthase (FX), is involved in the *de novo* synthesis of fucose and directly produces GDP-L-fucose which is the only donor of fucosylation. TSTA3-knockout mice exhibited a complete deficiency of fucosylation, confirming *de novo* pathway as the main source for cellular GDP-L-fucose 27. Our genomic data showed that TSTA3 was frequently amplified in ESCC and TSTA3 amplification was closely related to late stage and poor prognosis of ESCC patients. Although transcriptomics analysis in our cohort did not show significant transcript changes of TSTA3, there was a positive correlation between TSTA3 RNA expression and copy numbers, meanwhile TSTA3 protein was overexpressed in ESCC and its level was associated with ESCC progression. On the one hand, discrepancy between the abundance of protein and RNA molecules may be caused by the random error (e.g. sequencing platform, bioinformatics methods, sample size). On the other hand, other regulation such as post-transcriptional regulation, translation, post-translational processing may contribute to the fact that mRNA abundance correlates too weakly with protein abundance. Dynamic changes and overwhelmingly complex crosstalk among genomics, transcriptomics and proteomics may make the alteration in the protein level of TSTA3 more significant and useful to predict the clinical outcomes for ESCC.

It seemed that TSTA3 promotion of metastases in ESCC was independent of growth advantage conferred by TSTA3, since TSTA3 knockdown and overexpression did not impact the growth of ESCC. Consistent with the oncogenic role in our study, TSTA3 upregulation was reported in colorectal cancer, hepatocellular carcinoma, and breast cancer 28-30. In colorectal cancer, highly metastatic variants expressed higher levels of TSTA3 than low metastatic variants originating from the same tumor 31. In breast cancer, wild type TSTA3 exerted oncogenic effect and high expression of TSTA3 in tumor tissues was closely related to clinical stage and poor prognosis 28. In hepatocellular carcinoma, GEO data analysis showed that the TSTA3-related network included cell migration and invasion 32. TSTA3 was also found to be overexpressed in peripheral blood and tumor tissues of early stage lung adenocarcinoma patients 33. These findings indicated that TSTA3 was a promising marker and target for cancer diagnosis and therapy. However, in mouse model of colon cancer with a deletion of the TSTA3 locus, fucosylation deficiency led to suppressed Notch activation, occurrence of colitis and adenocarcinoma 34. In our study, the pro-metastasis role of TSTA3 in ESCC indicated that the level of TSTA3 enzyme affected cellular fucosylation and further altered the interaction of ESCC cells with some metastasis-related adhesion molecules. N-glycoproteomics analysis also revealed that communication among transmembrane protein and extracellular matrix, lysosome and phagosome-related proteins and cation-transporting ATPase activity etc. were involved in the TSTA3 mediated ESCC progression.

It is well known that there are two main types of N-fucosylation according to the location of fucose: core fucosylation and terminal fucosylation. The former one formed α-1,6-fucosylation, the main type of N-fucosylation which is under the charge of fucosyltransferases 8 (Fut8) transferring GDP-L-fucose to GlcNAc residue 35. The latter one formed α-1,2-fucosylation or α-1,3/4-fucosylation which is under the charge of Fut1-2 or Fut3-7/9-11 transferring GDP-L-fucose to terminal galactose or acetyl glucosamine residue 36. Moreover, core fucosylation and α-1,2-fucosylation can be specifically bound and recognized by LCA and UEA-I lectin respectively 37. In this study, LCA lectin analysis combined with N-glycoproteomics analysis identified core-fucosylated LAMP2, a major composition of the lysosomal membrane, underlying pro-metastasis effect of TSTA3 in ESCC 38. It has been reported that knockdown of FUT1 led to localization of LAMP2 from peripheral region to preferential perinuclear region, resulting in an increase of autophagosome formation 39. In fact, in aggressive cancer cells, lysosomes seemed to tend to shift from perinuclear to plasma membrane for release of contents facilitating migration/invasion of cancer cells 40-42. We found that LAMP2 also had core fucosylated modification in ESCC. Whether abnormal core fucosylation of LAMP2 participate in the TSTA3-mediated ESCC metastasis through abnormal localization of lysosomes needs to be further studied. Consistently, in both breast cancer and ovarian clear cell adenocarcinoma, reduced expression of LAMP2 significantly inhibited the metastasis of cancer cells 43, 44.

UEA-I lectin analysis combined with N-glycoproteomics analysis also identified terminal fucosylated ERBB2 underlying pro-metastasis effect of TSTA3 in ESCC. It is well known that overexpression of ERBB2 contributes to the metastatic cascade in many types of cancers including ESCC. ERBB2 activity, interaction with growth factors and adhesion molecules, may be altered by terminal fucosylation. It has been reported that EGFR had potential N-glycosylation sites, more than half of which were modified by fucosylation 45. N-glycosylation inhibitors significantly reduced the binding of EGF to EGFR, and core fucosylation EGFR had a higher affinity with EGF 46. Although fucosylation of ERBB2 has not been reported in cancer, there was evidence supporting that the antibody-dependent cellular cytotoxicity activity of the de-fucosylated antibody of ERBB2 significantly increased compared to the wild-type antibody 47, 48.

Combining our experimental results, there may be two reasons for the increased core fucosylated LAMP2 and terminal fucosylated ERBB2 mediating pro-invasion of TSTA3 in ESCC. One may be that TSTA3 amplification and overexpression lead to the increase of GDP-Fucose pool through raising the conversion of GDP-mannose to GDP-fucose, which was confirmed by the lectin immunofluorescence assay in TSTA3 knockdown and overexpressed ESCC cells in this study. The other reason may be the increased enzymatic activity of fucosyltransferases, which was verified by glycosyltransferase activity assay, lectin affinity enrichment followed by western blot and immunoprecipitation followed by lectin blot in this study. In support of this view, the effect of TSTA3 ectopic expression on promoting invasion could be mostly attenuated by silencing of LAMP2 and ERBB2. Moreover, there may be a synergistic effect of LAMP2 and ERBB2 in TSTA3 mediated pro-invasion in ESCC. Meanwhile, the integrated omics analysis and functional experiment indicated that it was an increase in the proportion of fucosylated LAMP2 and ERBB2 rather than the increase of total protein expression that triggered ESCC metastasis.

Combined with the results that upregulated fucosylation level was required for cancer growth and metastasis 49, 50, our results highlighted the therapeutic potential of targeting TSTA3 and fucosylation inhibitor to treat metastatic ESCC. As expected, treatment with peracetylated 2-F-Fuc in ESCC effectively suppressed cell invasion which is consistent with studies in hepatocellular carcinoma 51. Indeed, 2-F-Fuc treatment provided complete protection against tumor engraftment in the tumor vaccine mouse model and has been developed for clinical application 52. As a fucosyltransferase inhibitor, 2-F-Fuc also reduced the expression of TSTA3 in ESCC, which might be attributed to the fact that the accumulation of GDP-2F-Fuc through salvage pathways shut down the *de novo* synthesis of GDP-Fucose via a feedback loop 50. 6-alkynyl-fucose (6-Alk-Fuc), which is another fucose analog and depletes the cellular GDP-Fucose pool directly targeting TSTA3, exhibited much higher potency than 2-fluorofucose, and suppressed cancer cell migration *in vitro* 53. Furthermore, fucose-liposome nanoparticles have been developed as a targeted antitumor drug carrier and have been shown to efficiently inhibit *in vivo* growth of colorectal tumors 54.

In summary, our study provides evidences supporting that TSTA3 harbors frequent copy number amplification and overexpression, which are associated with late stage and poor prognosis in ESCC. Moreover, TSTA3 may play a role in promoting invasion through increase fucosylation levels of LAMP2 and ERBB2 in ESCC. We believe that fucosylation inhibitor targeting TSTA3 shows promise as an effective compound in ESCC therapy.

## Materials and Methods

### Samples and clinical data

A total of 508 ESCC patients with FFPE samples used for WGS were recruited from Shanxi and Xinjiang provinces in China. The description of the clinical characteristics of the 508 samples was presented in our previous study 21. A total of 155 ESCC patients with fresh frozen tissues used for both WGS and transcriptomic sequencing were recruited from Shanxi provinces in China. The fresh tissues were frozen into liquid nitrogen within 30 min post-surgery. All individuals had received no prior treatment and gave their informed consent. The study was approved by the ethical committees of the Shanxi Medical University. Each tumor specimen had paired adjacent, histologically normal tissues. All cases were classified according to WHO criteria. Hematoxylin and eosin (H&E)-stained sections from each sample were subjected to review by at least three independent pathologists. The ESCC individuals were staged according to the Cancer Staging Standards of the American Joint Committee on Cancer (eighth edition, 2017).

A human ESCC tissue array containing 104 primary ESCC tissues and 60 paired normal tissues was used as previously 21. Meanwhile, we collected paraffin-embedded samples from 11 ESCC patients including primary ESCC, matched non-tumor tissues and metastatic lymph node (1-3 samples per patient). All patients signed their own informed consent and all samples were obtained before treatment according to the guidelines of the local ethical committees (IRB of Shanxi Medical University, Approval No. 2017LL037). Details of clinicopathological features were summarized in **Table S1** and **S3.**

### Whole genomic sequencing (WGS)

The WGS was performed as described previously 21. In brief, after DNA extraction, Sample Purification Beads (Illumina) was used to purify fragmented DNA. Adapter-ligated libraries were prepared with the TruSeq Nano DNA Sample Prep Kits (Illumina). Illumina cBOT cluster generation system with HiSeq PE Cluster Kits (illumina) was used to generate clusters. Paired-end sequencing was performed using an Illumina HiSeq system following Illumina's instructions in WuXi NextCODE at Shanghai, China. For WGS of 508 FFPE samples, the mean sequencing coverages were 98× and 44× for tumor tissues and matched normal samples, respectively. Sequence coverage exceeding 20× was 98.0% for tumors and 92.6% for normal samples 21. For WGS of 155 FFPE samples, the mean sequencing coverages were 90× and 39× for tumor tissues and normal tissues respectively. Sequencing coverage exceeding 20× for tumor and normal were 97.52% and 89.22% respectively.

### RNA-sequencing

Total RNAs were isolated from tissue samples using the TRIzol reagent (Life Technologies, Carlsbad, CA, USA). mRNA was isolated from total RNA using the oligo-dT magnetic beads and braked into fragments for cDNA libraries construction. After the quality inspection, cDNA libraries were sequenced on WuXi NextCODE Genomics (Shanghai) Co., Ltd., China. Raw reads were subjected to quality control, filtered into clean reads and then aligned to the reference sequences. The alignment data were utilized to calculate distribution of reads on reference genes and mapping ratio. The total number of sequenced reads ranged from 30 million to 180 million pairs, and an average 94.3% of the pairs were aligned to the hg19 genome assembly using the STAR aligner. The percentage of genomic alignment was similar between the tumor and non-tumor tissues (mean ± standard deviation: 93.82 ± 4.2% and 94.82 ± 2.2%, respectively), suggesting no obvious detectable biases in the sequence data.

### Copy number analysis

To detect DNA copy-number alterations (CNAs), we used BIC-seq2 55 to analyze somatic copy number variations in ESCC genomes on the basis of WGS reads. Somatic CNAs were called by BIC-seq2-seg by comparing the normalized tumor and normal data. Copy numbers of ≤ 1.5 were considered as deletions and ≥ 2.5 were considered as amplifications. To infer recurrently genomic regions, we re-implemented GISTIC algorithm 56 using copy numbers in 1-kb windows as markers. G-scores were calculated for genomic and gene-coding regions based on the frequency and amplitude of amplification or deletion affecting each gene. A significant CNA region was defined as having amplification or deletion with G-score > 0.1, corresponding to a *P*-value threshold of 0.05 from permutation-derived null distribution.

### Immunohistochemistry

The sections were deparaffinized and rehydrated with xylene and gradient alcohol respectively. Then the sections were soaked with 3% H_2_O_2_ for 15 min. Antigen retrieval was implemented in sodium citrate buffer (pH 6.0) or Tris-EDTA buffer (pH9.0) for 4 min in a pressure cooker. After antigen retrieval and blocking, slides were incubated with the TSTA3 antibody (Abcam, Cat# ab190002) at a 1:200 dilution overnight at 4 °C, followed by detection using the PV8000 (Zhongshan, Beijing, China) and DAB detection kit (Maixin, Fuzhou, China). The protein expression level was analyzed using fully automatic digital pathological scanning apparatus (Aperio, Vista, CA, USA). Immunoreactivescore (IRS) was automatically generated by Aperio Cytoplasma 2.0 software and calculated by assessment of both the staining intensity and the percentage of positive cells.

### Knockdown and overexpression of genes of interest in ESCC lines

Plasmid constructs and lentiviral packaging were performed in Shanghai GenePharma Co., LTD. For knockdown of endogenous TSTA3, lentiviral virus shRNA sequences were cloned into the pGLV-H1-GFP-Puro vector and co-transfected into HEK-293 cells along with packaging plasmids. To lentivirus transduction, ESCC cells were seeded at 30%-40% confluence and infected with suitably titered viral supernatant (1.5 ml/well) and incubated overnight. Then, the viral supernatant was replaced with fresh media and cells were selected by puromycin (2 µg/ml) (Invitrogen; Thermo Fisher Scientific, Inc.) after 48 h. For knockdown of endogenous LAMP2 and ERBB2, three siRNAs (Guangzhou RiboBio) were used. All the siRNAs sequence and primers for Real-time qPCR used in this study were included in **Table S5**. For overexpression of TSTA3, we purchased viruses from Shanghai Genechem Co., LTD and the cDNA of wild-type TSTA3 gene was cloned into the lentivirus vector of EF-1aF/Luciferase05&puro and targeting ESCC cells were infected with MOI (multiply of infection) values of 150.

### Western blotting

The cells were lysed in RIPA buffer (1% Triton X-100, 50 mM Tris-HCl, pH 7.6, 150 mM NaCl, 1% sodium deoxycholate, and 0.1% SDS) with protease and phosphatase inhibitors (Thermo Fisher Scientific) on ice and centrifuged at 4 °C at 12,000 rpm for 15-25 min. The concentrations of supernatant were determined by Bradford method. Equal amounts of protein were loaded and separated by 10% SDS-PAGE and transferred onto PVDF membranes. After blocking, the membrane was incubated with the dilution of the primary antibody overnight at 4 °C as follows: β-actin (proteintech, Cat# 60008-1-Ig, 1:5,000), GAPDH (proteintech, Cat# 60004-1-Ig, 1:5,000), TSTA3 (Abcam, Cat# ab190002 and ab155306, 1:500), LAMP2 (Abcam, Cat# ab199946, 1:1000) and ERBB2 (Abcam, Cat# ab134182, 1:1000). Antibody binding was detected with horse radish-peroxidase-conjugated anti-mouse (Sigma) or anti-rabbit (Cell Signaling) antibodies for 2 h at room temperature.

### Invasion and migration assay

The invasion and migration ability of cells were detected using transwell plates (8 µm, Corning, Inc.), as described in our previous study 57. For the migration assay, 50,000~80,000 cells were seeded into each well with serum-free medium in the upper compartment of the transwell plates. The lower compartment of the chamber was filled with medium with 10% FBS. After being cultured for 24 h or 48 h, the cells on the upper surface were removed; the cells that passed though the membrane were fixed with 4% formaldehyde and stained using 0.1% crystal violet. Random five fields were chosen to count the number of transmigrated cells. For the invasion assays, the upper chambers were pre-coated with 100 μl of Matrigel (1: 8 mixed with FBS-free media; BD Biosciences, Heidelberg, Germany) and proceeded using the same as described above. Each experiment consisted of thee replications and was repeated at least three times. For fucosylation inhibitor assay, the peracetylated 2-fluoro 2-deoxy-L-fucose was purchased from Biosynth Carbosynth (Cat#: MT15919). The optimum working concentration was obtained by preliminary experiment.

### MTT and colony formation assay

5×10^3^ cells were seeded into each well of a 96-well plate in a final volume of 200 µl conditioned media. At different time points, 20 µl of 5 mg/ml MTT (Invitrogen, USA) was added into each well and the cells were incubated for 4 h at 37 °C. After solution was removed, 200 µl of DMSO was added to dissolve the crystals. The absorbance was measured with a Spectrophotometer at 490 nm. For colony formation assay, the cells were seeded at a density of 500-800 cells/well in 6-well plate and incubated at 37 °C and 5% CO2 for 15 days. Then cells were fixed with 4% polyformaldehyde for 15 min and stained with 1% crystal violet and the numbers of colonies containing more than 50 cells were counted microscopically.

### Apoptosis assay

Cell apoptosis was assessed using an Annexin V‑APC/7‑AAD Apoptosis Detection kit (KeyGen Biotech Co., Ltd., Nanjing, China) according to the manufacturer's protocol. Briefly, cells were re-suspended in 500 µl binding buffer, 5 µl Annexin V‑APC and 5 µl 7‑AAD, followed by being incubated at room temperature for 30 min in the darkness. Subsequently, the cells were analyzed by flow cytometry (FACS-Calibur; BD Biosciences, Franklin Lakes, NJ, USA).

### Flow cytometry analysis

For the assay of flow cytometry, 1-2×10^5^ cells were seeded per well in 6-well plates containing complete medium. After cell adhesion, 10 μg FITC-UEA (Sigma) or Fluorescein labeled LCA (Vector lab) and appropriate amount of PBS were added to each well until the total volume was 1000 μl. After being gently mixed, the samples were incubated at 37 °C in darkness for 1 h. Then, the cells were detached with 0.2% trypsin in darkness and the re-suspended single-cell suspensions were fixed with 1% paraformaldehyde for 20 min. The fluorescence intensity was analyzed using a FACS Calibur machine (BD).

### Label-free quantitative proteomics

Samples were lysed using four volumes of lysis buffer (8 M urea, 1% Protease Inhibitor Cocktail) and subsequently sonicated on ice using a high intensity ultrasonic processor (Scientz). After removing the debris, dithiothreitol was added to a final concentration of 5 mM and the protein solution was reduced for at 56 °C, 30 min. Then, iodoacetamide was added to a final concentration of 11 mM and the samples were incubated for 15 min at room temperature in darkness. Finally, trypsin was added at 1:50 trypsin-to-protein mass ratio for the first digestion overnight and 1:100 trypsin-to-protein mass ratio for a second 4 h digestion. For HPLC fractionation, the tryptic peptides were fractionated into fractions by high pH reverse-phase HPLC using Agilent 300Extend C18 column.

### N-glycoproteomic quantification analysis

Tryptic peptides were dissolved in 40 µl of enrichment buffer (80% acetonitrile/1% trifluoroacetic acid). The supernatant was transferred to a hydrophilic (HILIC) micro-column and centrifuged at 4000 g for approximately 15 min to complete enrichment. Then the hydrophilic micro-column was washed and the glycopeptide was then eluted with 10% acetonitrile, and the eluate was collected and vacuum dried. The samples were re-dissolved in 50 mM ammonium bicarbonate buffer and digested by adding 2 μl of PNGase F glycosidase at 37 °C overnight. Then, the resulting peptides were desalted with C18 ZipTips (Millipore) according to the manufacturer's instructions.

All LC-MS/MS analysis and bioinformatics analysis were completed by Jingjie PTM Biolabs Inc (Hangzhou, China). The peptides were dissolved in 0.1% formic acid (solvent A) and directly loaded into a home-made reversed-phase analytical column. The gradient consisted of a gradual. They all operated at a constant flow rate of 400 nL/min on EASY-nLC 1000 UPLC system. The peptides were submitted to NSI source and were analyzed by tandem mass spectrometry (MS/MS) in Q ExactiveTM Plus (Thermo) coupled online to the UPLC. The electrospray voltage applied was 2.0 kV. The m/z scan range was 350-1550 for full scan, and Orbitrap was used to detect intact peptides at a resolution of 60,000. Peptides were then selected for MS/MS using NCE setting as 28 and the fragments were detected in the Orbitrap at a resolution of 15,000. A data-dependent procedure that alternated between one MS scan followed by 20 MS/MS scans with 15.0 s dynamic exclusion. Automatic gain control (AGC) was set at 5E4. Fixed first mass was set as 100 m/z.

### LCA and UEA-I affinity chromatography

For lectin enrichment assays, the cells were lysed with lysis buffer containing protease inhibitor and 1% Nonidet P-40. 100 µl of biotinylated LCA (Vector lab, Cat# B-1045) or UEA-I (Vector lab, Cat# B-1065) lectin was added to above 1000 µg of lysate and volume was made up to 1000 µl with phosphate buffered solution (PBS) containing 1 mM MnCl2, 1 mM MgCl2, and 1 mM CaCl2. The mixture was incubated at 4 °C overnight with rotation and then, 60 µl of prewashed agarose coupled streptavidin (Vector lab, Cat# SA-5010) was added and incubation was continued for another 4 h. The beads were washed after centrifugation and subsequently separated with 10% SDS-PAGE after boiling at 95 °C for 5 min. The samples were transferred to nitrocellulose filter membranes and then the membranes were incubated with special antibodies.

### Extraction of fucosylated protein

After affinity chromatography as above, the proteins containing the corresponding fucose chains can be adsorbed. The enriched glycoproteins were firstly eluted by eluent containing 0.15 mol/L NaCl, 10 mmol/L Tris-HCl (pH = 2.0) to A280 < 0.001. Then the samples were eluted by 100 mM α-L-fucose. After dialysis and lyophilization desalination, protein concentration was determined using bicinchoninic acid (BCA) protein assay kit (Pierce, Rockford, IL, USA). Protein concentration was adjusted to 0.02 mg/ml as the working concentration and was used for transwell assay. Alternatively, samples were treated with fucosidase (NEB) according to the manufacturer's protocol at 37 °C for 4 h.

### Immunoprecipitation

Cells were lysed (lysis buffer: 50 mM Tris, 150 mM NaCl, 2 mM EDTA, 1 mM phenylmethylsulfonyl fluoride-PMSF, 0.5% Triton X-100) and incubated for 1 h on ice. Cell lysate was centrifuged 30 min at 4 °C and an aliquot of the supernatant was separated as input. 1000 µg of cell lysate, 2-5 µg of primary antibody including LAMP2 and ERBB2 and 50 µl ready-to-use protein G-agarose beads were incubated at 4 °C overnight. Next day, the pellet was collected, washed and finally re-suspended in the SDS sample buffer. After being boiled for 10 min, the immunoprecipitated complexes were released for western blot analysis. Treatment with PNGase F (NEB) was performed as described by manufacturer. For lectin blotting, samples were incubated with biotinylated LCA and UEA-I lectin (5 µg/ml) in TBST at 4 °C overnight and then incubated with HRP-conjugated streptavidin (0.2 µg/ml) at room temperature for 2 h.

### *In vivo* experiments

The use of experimental animals was approved by The Ethics Committee of Shanxi Medical University and strictly followed the guideline for tumor induction in mice and rats. All mice (Vital River Laboratory Animal Technology Co., Ltd., Beijing, China) were kept in standard, pathogen-free conditions under a 12-h light/dark cycle with 22-26 °C temperature and 45-65% humidity and free access to food and water. Two independent experiments were carried out in KYSE150 cell and five mice in each group for both experiments. Metastatic tumor model was established by giving intravenous tail vein injections of 2.0×10^6^ cells/mouse to two groups of mice at age of 4-6 weeks. After 6 weeks, the mice were sacrificed by excessive injection of 2% sodium pentobarbital, and the number of metastatic nodules on the lung and liver surfaces were counted under a dissecting microscope at ×10 magnification. The tumors were embedded in paraffin for further study. All animal studies were conducted with the approval of the Shanxi Medical University Institutional Animal Care and Use Committee.

### Enzyme activity assay

The fucosyltransferase activity was measured by the Glycosyltransferase activity kit (EA001, R&D system/USA). This kit utilizes the coupling phosphatase to remove inorganic phosphate quantitatively from the leaving nucleotide diphosphate (such as GDP) generated during glycosyltransferase reactions. The released inorganic phosphate is then assayed by Malachite Green phosphate detecting reagents and the fucosyltransferase activity is reflected by the rate of inorganic phosphorus production in a given amount of protein solution. The activity of FUT8 was measured by its ability to hydrolyze the donor substrate GDP-L-Fucose (sc-221696, Santa Cruz) and FUT2 by its ability to transfer fucose from GDP-L-Fucose to alpha-lactose (L2643, Sigma). The reaction conditions and detailed method was according to the manufacturer's protocol.

### Statistical analysis

Statistical analyses were performed using SPSS 22 software package and GraphPad Prism 6. Univariate binary logistic and Chi square (χ^2^) tests were used to analyze the association of TSTA3 amplification and expression with clinicopathological features. Survival analysis was carried out using Kaplan-Meier analysis and log-rank test. All experiments were done in triplicates and data were presented as mean ± SD. Student's t-test was used for statistical analysis, and data from more than two groups were analyzed by one-way analysis of variance (ANOVA) followed by Dunnett's test. The correlation between TSTA3 amplification and TSTA3 mRNA expression level was analyzed using Pearson correlation analysis.

## Supplementary Material

Supplementary figures and tables.

## Abbreviations

TSTA3: Tissue Specific Transplantation Antigen P35B/GDP-4-keto-6-deoxymannose-3,5-epimerase-4-reductase
ESCC: esophageal squamous cell carcinoma
2-F-Fuc: 2-fluoro 2-deoxy-L-fucose 2-fluorofucose
AFP: alpha-fetoprotein
Fuc-Hpt: fucosylated haptoglobin
WGS: whole-genomic sequencing
FFPE: formalin-fixed paraffin-embedded
FCNA: focal copy number alterations
HR: hazard ratio
CI: confidence interval
FX: GDP-fucose synthase
TCGA: The Cancer Genome Atlas
CCLE: Cancer Cell Line Encyclopedia
qPCR: quantitative real-time PCR
NC: negative control
WT: wild-type
GO: Gene Ontology
KEGG: Kyoto Encyclopedia of Genes and Genomes
LAMP2: lysosomal associated membrane protein 2
IKBIP: IKBKB interacting protein
LMNA: lamin A/C
ALCAM: activated leukocyte cell adhesion molecule
SLC39A14: solute carrier family 39 member 14
GAA: glucosidase alpha, acid
IP: immunoprecipitation
ERBB2: erb-b2 receptor tyrosine kinase 2
Fut8: fucosyltransferases 8
IRS: immunoreactivescore
siRNA: small interference RNA
MOI: multiply of infection
HILIC: Hydrophilic Interaction Liquid Chromatography
PBS: phosphate buffered solution
BCA: bicinchoninic acid
CBB: Coomassie brilliant blue
