# Supplementary material for: TSTA3 facilitates esophageal squamous cell carcinoma progression through regulating fucosylation of LAMP2 and ERBB2
Source: Theranostics. 2020 Sep 14;10(24):11339–58. doi: 10.7150/thno.48225 (PMC7532669; doi:10.7150/thno.48225)
Supplement: Supplementary file 1 — Supplementary figures and tables. [file thnov10p11339s1.pdf]

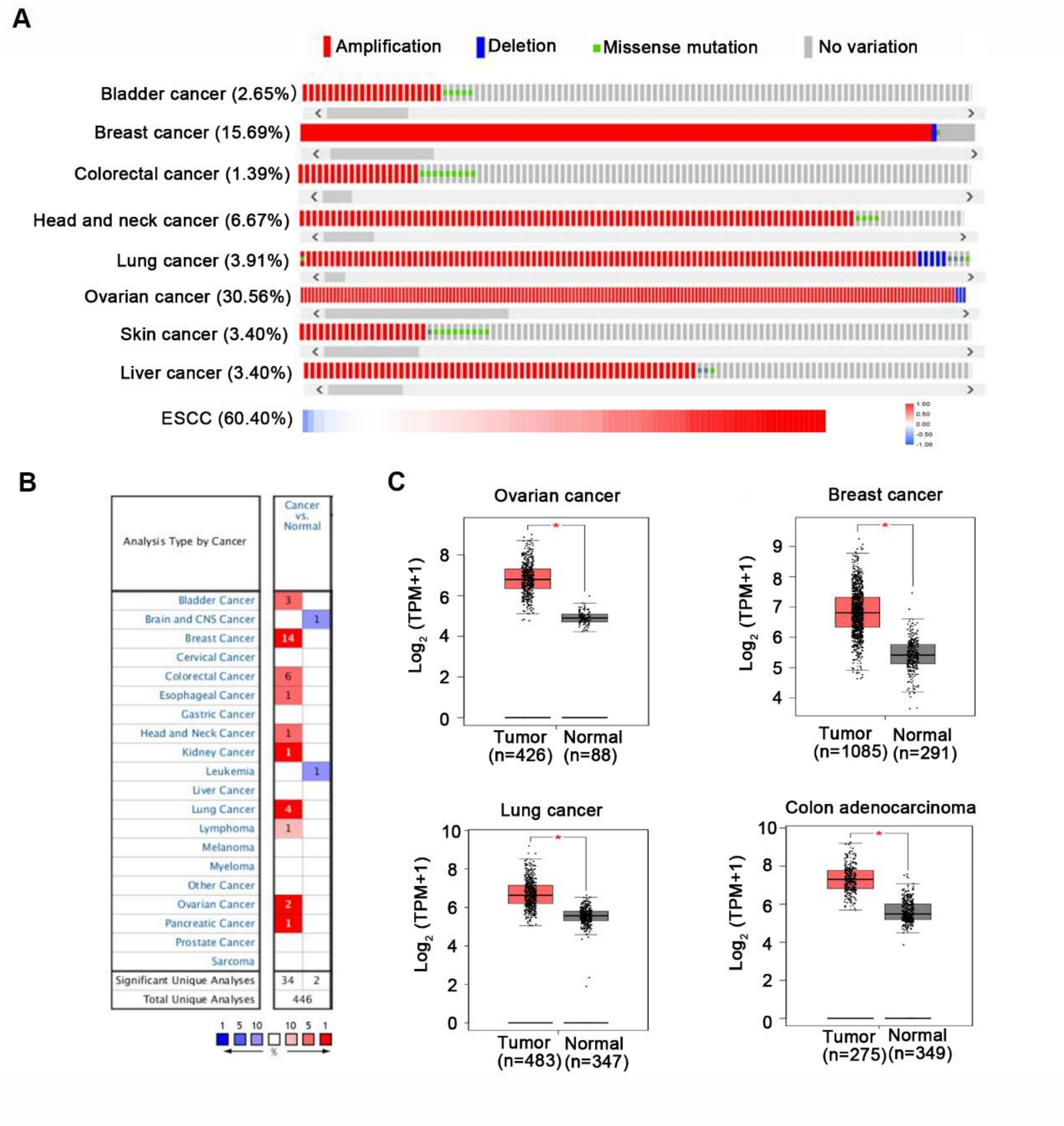

Figure S1. Amplification and expression analysis of TSTA3 in TCGA database. (A) Amplification and variation of TSTA3 gene in different cancers. **(B)** Expression level of TSTA3 in different cancers. Red represents the expression of TSTA3 in cancer tissue is higher than that of normal tissue. Blue represents the expression of TSTA3 in cancer tissue is lower than that of normal tissue. **(C)** In ovarian cancer, breast cancer, lung cancer and colon adenocarcinoma TSTA3 mRNA was dramatically upregulated in tumor tissues in comparison to that of non-tumor tissues in TCGA database.

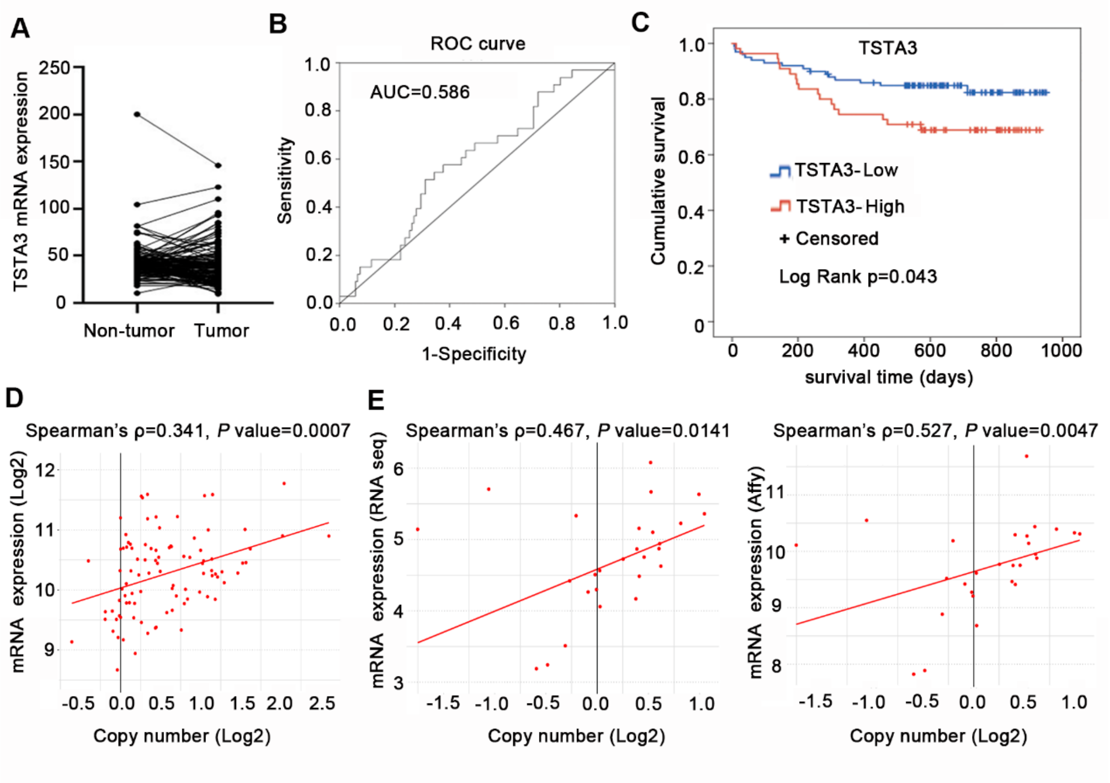

Figure S2. The expression analysis of TSTA3 in transcriptomic sequencing data of ESCC. (A) The expression level of TSTA3 mRNA in ESCC tissues compared with paired normal tissues from 155 cases. **(B)** ROC curve analyses of TSTA3 mRNA expression in RNA sequencing data of 155 ESCC cases (AUC = 0.586, $P < 0.001$). **(C)** Kaplan-Meier's analysis between ESCC patients with different TSTA3 mRNA expression in RNA sequencing data of 155 ESCC cases. **(D)** Correlation analysis between TSTA3 amplification and RNA expression in ESCC tissues of TCGA database. **(E)** Correlation analysis between TSTA3 amplification and RNA expression in 27 ESCC cell lines of Cancer Cell Line Encyclopedia (CCLE) database.

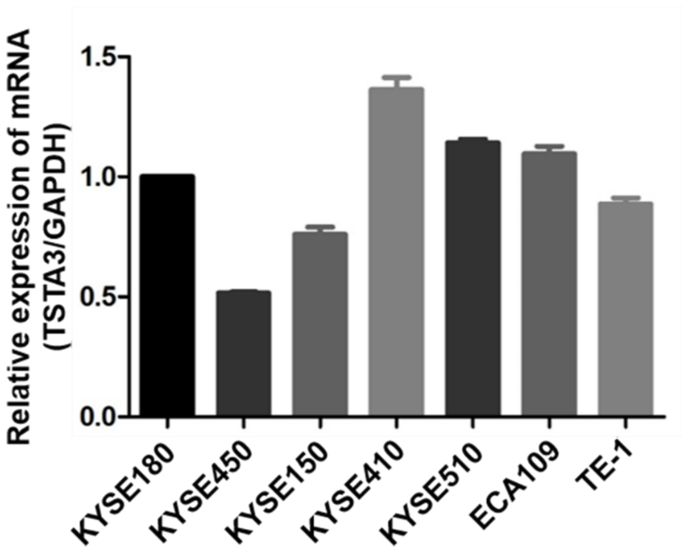

Figure S3. The mRNA expression pattern of TSTA3 in ESCC cell lines detected by RT-qPCR.

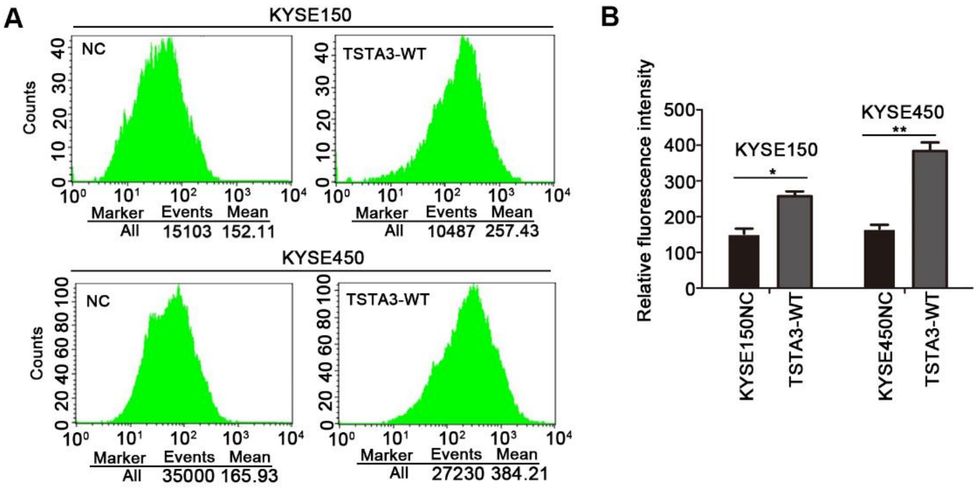

Figure S4. UEA-I fluorescence analysis of ESCC cells. (A) UEA-I fluorescence of KYSE150 (upper) and KYSE450 (bottom) cells stably overexpressing TSTA3 or control vector were analyzed by FACS. **(B)** The panel shows quantitation of fluorescence. All data are presented as the mean ± standard deviation and three independent experiments. *$P < 0.05$, **$P < 0.01$.

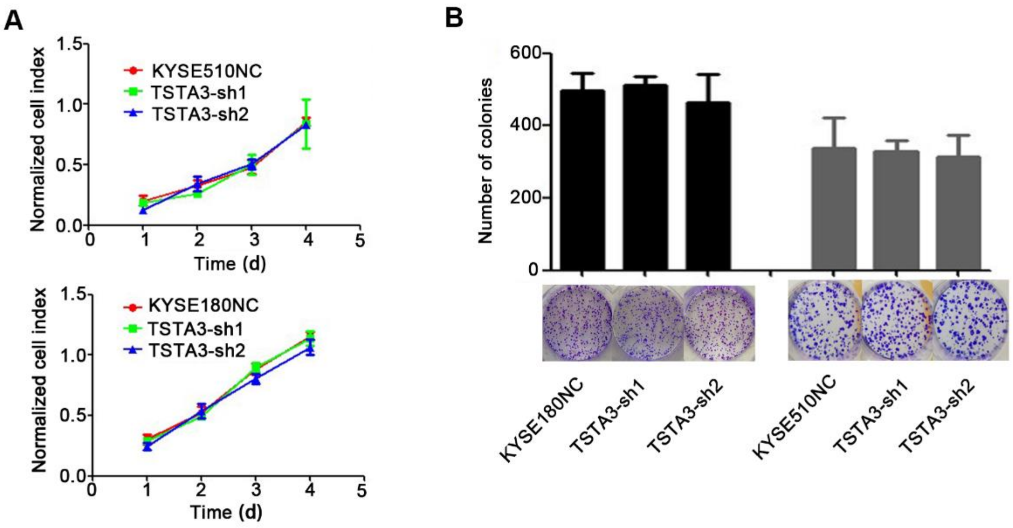

Figure S5. The effect of TSTA3 silencing on ESCC proliferation and colony formation. (A) Cell proliferation ability of KYSE510 (upper) and KYSE180 (bottom) cells with TSTA3 knockdown and negative control was analyzed by MTT. **(B)** The ability of colony formation was analyzed by colony formation assay. All data are presented as the mean ± standard deviation and three independent experiments.

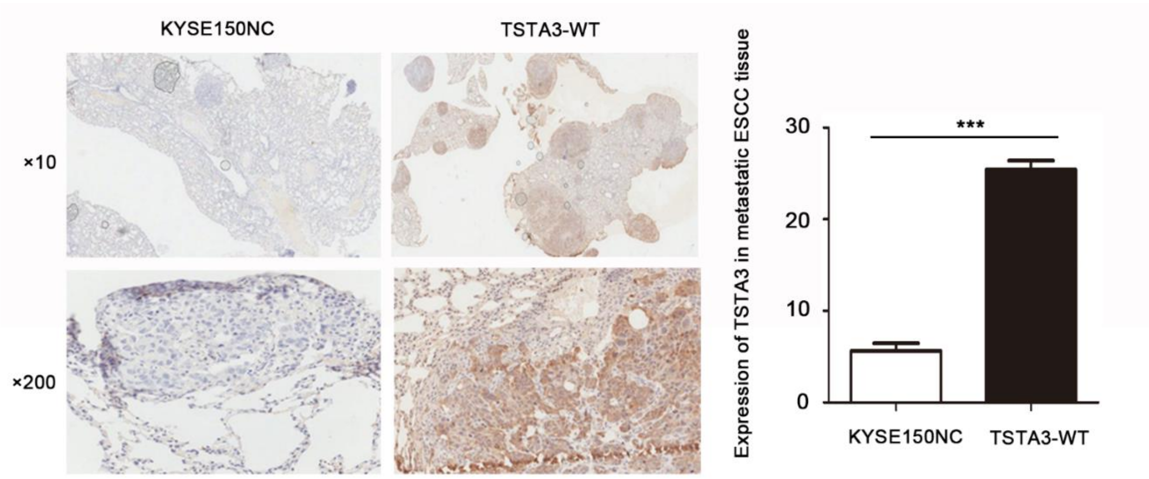

Figure S6. Representative immunohistochemistry images of TSTA3 expression in metastatic tumor nodules in the lung section of nude mice intravenously injected with KYSE150 cells stably overexpressing TSTA3 or control vector.

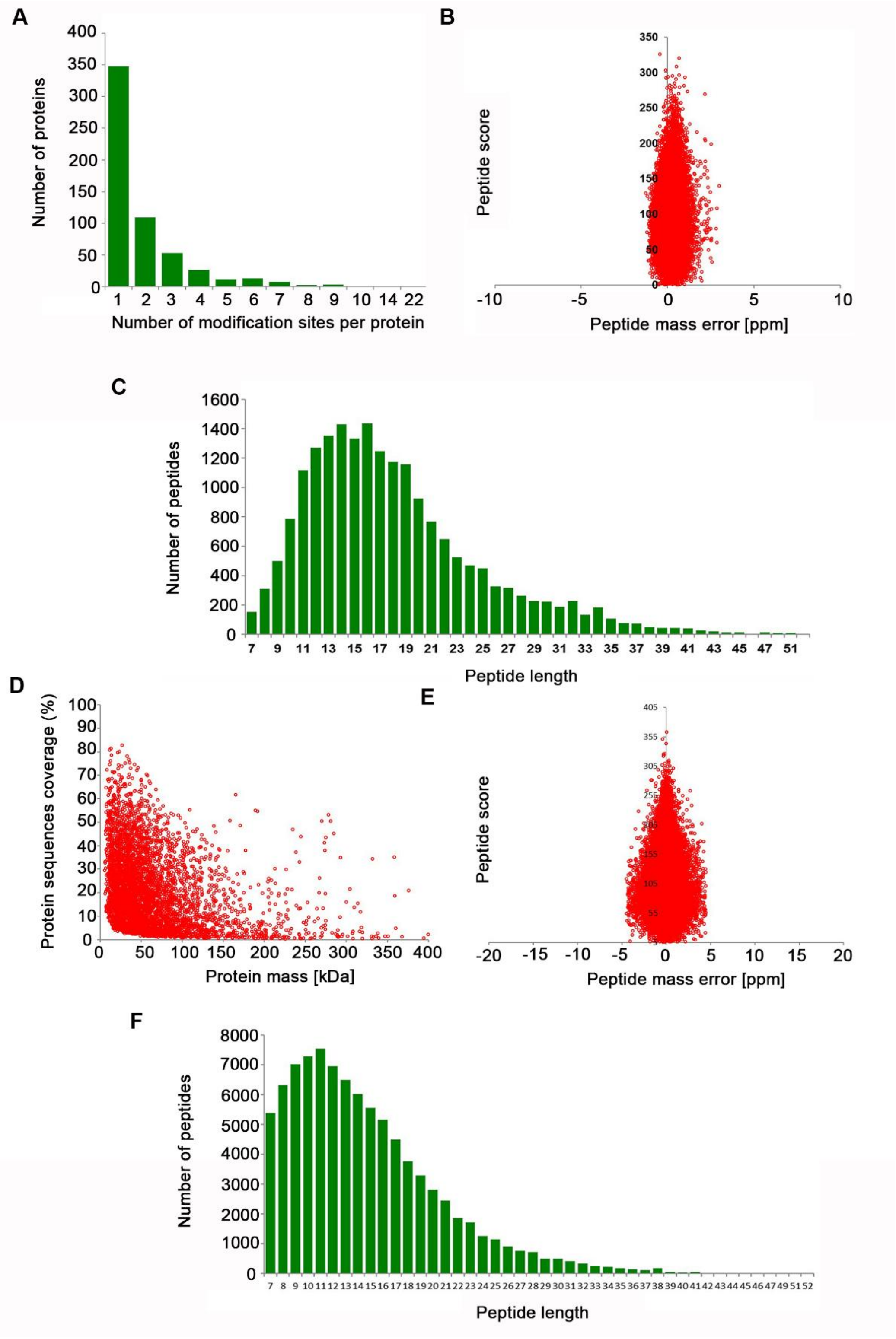

Figure S7. Quality control of N-glycoproteomics and proteomics analysis. (A) Statistic analysis of the number of modified site per glycosylated proteins. **(B)** The mass error of the whole identified peptides in N-glycoproteomics analysis. **(C)** Statistic analysis of the whole identified peptide length in N-glycoproteomics analysis. **(D)** The relationship between protein coverage and molecular weight in proteomics analysis. **(E)** The mass error of the whole identified peptides in proteomics analysis. **(F)** Statistic analysis of the whole identified peptide length in proteomics analysis.

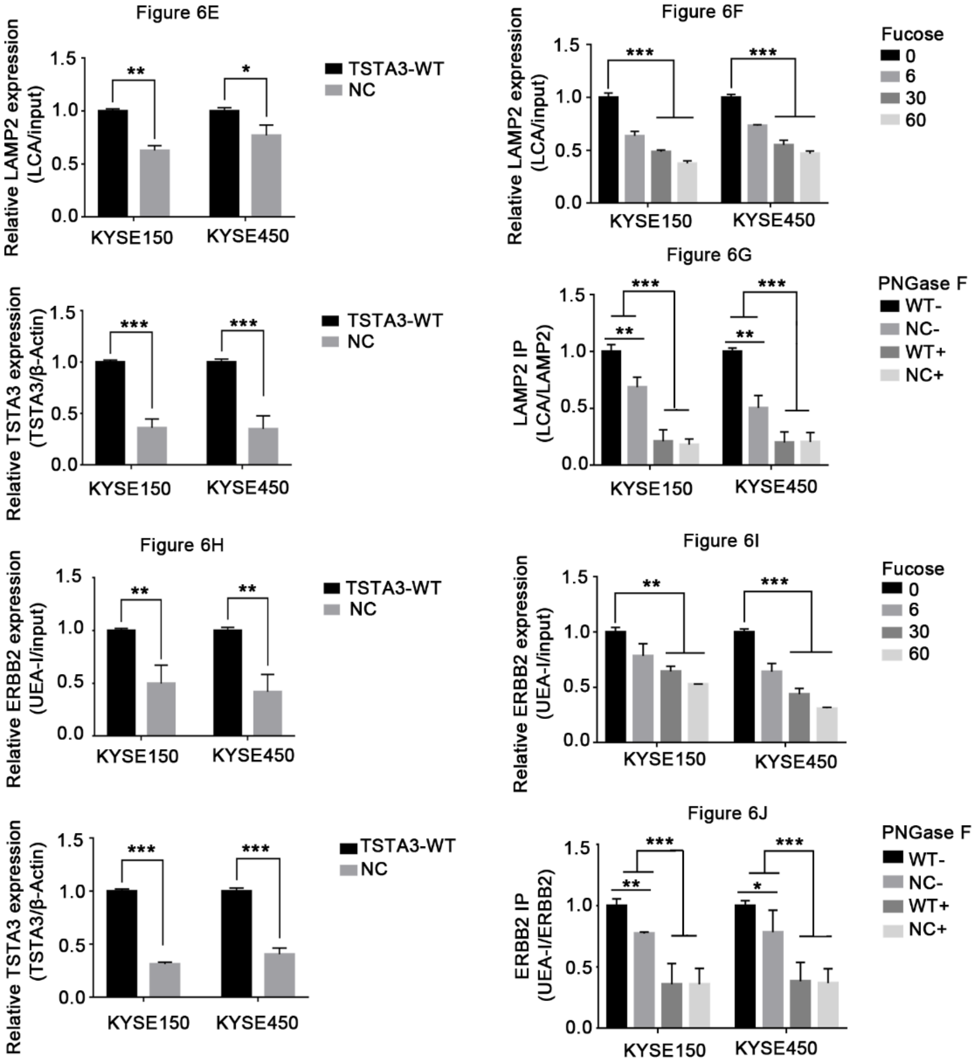

Figure S8. The densitometries analysis on the western blots of Figure 6E-6J. ***$P < 0.001$, **$P < 0.01$, *$P < 0.05$.

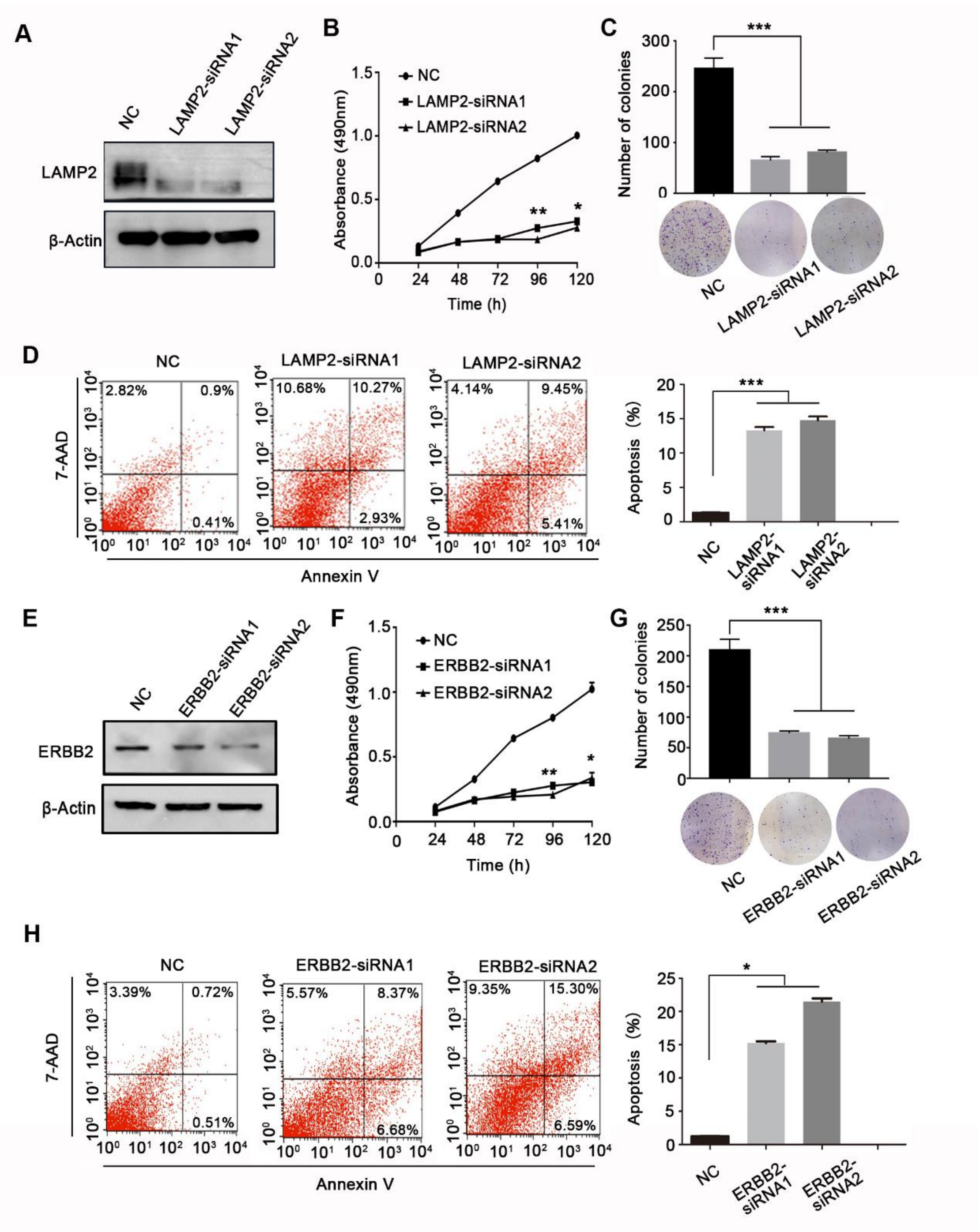

Figure S9. The effect of LAMP2 or ERBB2 knockdown on the proliferation, colony formation and apoptosis of KYSE150 ESCC cells. (A, E) Knockdown efficiency of LAMP2 or ERBB2 were tested by western blot. **(B, F)** LAMP2 or ERBB2 knockdown inhibited the proliferation of KYSE150 cells monitored by MTT assay. **(C, G)** The numbers of colony formation were decreased in LAMP2 or ERBB2 knockdown group compared to the control. **(D, H)** LAMP2 or ERBB2 knockdown increased apoptosis rate of KYSE 150 cells. Data represent the mean ± SD. At least three independent experiments were performed in triplicate. Statistical analysis was performed with a two-sided t test. ***$P < 0.001$, **$P < 0.01$, *$P < 0.05$.

Table S1: Association of TSTA3 amplification with clinic-pathologic factors in 663 ESCC cases.

Clinical, epidemiological or pathological feature		Total(N)	TSTA3_WT	Proportion	TSTA3_AMP	Proportion	*P* value
All cases		663	438	66.06%	225	33.94%	
Age	<60	275	170	61.82%	105	38.18%	0.13178
	60-69	301	210	69.77%	91	30.23%	
	≥70	87	58	66.67%	29	33.33%	
Sex	Male	438	284	64.84%	154	35.16%	0.353595
	Female	225	154	68.44%	71	31.56%	
Tumor location	Upper thoracic	34	17	50.00%	17	50.00%	0.126173
	Middle thoracic	427	288	67.45%	139	32.55%	
	Lower thoracic	202	133	65.84%	69	34.16%	
Histological grade	Grade 1	42	30	71.43%	12	28.57%	0.619902
	Grade 2	395	256	64.81%	139	35.19%	
	Grade 3	226	152	67.26%	74	32.74%	
Pathologic Stage	I&II	383	271	70.76%	112	29.24%	0.002926
	III&IV	280	167	59.64%	113	40.36%	
Lymphatic.metastasis	Yes	283	168	59.36%	115	40.64%	0.001734
	No	380	270	71.05%	110	28.95%	
Prognosis (Log-rank Mantel-Cox test)	Dead	251	165	65.74%	86	34.26%	0.877426
	Survival	408	270	66.18%	138	33.82%	
	Missing	4	3	75.00%	1	25.00%	

Table S2: Association of TSTA3 mRNA expression with clinic-pathologic factors in 155 ESCC cases

Characteristics		Total (n=155)	TSTA3 expression n(%)		*P* value
			Low	High	
Age	<60	56	32(57.1)	24(42.9)	0.149
	≥60	99	68(68.7)	31(31.3)	
Sex	Female	52	34(65.4)	18(34.6)	0.872
	Male	103	66(64.1)	37(35.9)	
Tumor location	Upper and Middle	39	25(64.1)	14(35.9)	0.095
	Lower	116	75(64.7)	41(35.3)	
Histological grade	Grade 1	4	3(75.0)	1(25.0)	0.468
	Grade 2	105	67(63.8)	38(36.2)	
	Grade 3	42	26(61.9)	16(38.1)	
	Grade 4	4	4(100.0)	0(0.0)	
Pathologic Stage	I&II	83	59(71.1)	24(28.9)	0.067
	III&IV	72	41(56.9)	31(43.1)	
Pathologic T Stage	1+2	41	24(58.5)	17(41.5)	0.351
	3+4	114	76(66.7)	38(33.3)	
Lymphatic.metastasis	No	82	58(70.7)	24(29.3)	0.087
	Yes	73	42(57.5)	31(42.5)	

Table S3. Clinical features of ESCC patients having metastatic lymph nodes samples

Sample ID	gender	Age at diagnosis	Tumor Grade	location(Upper/ Middle/Lower)	Smoking Status	Drinking Status	Pathological T	Pathological N	Clinical M	Stage(TNM)	Lymphatic metastasis	Lymph node	Survival status	Survival after diagnosis (months)
ESCC007	Female	66	G3	Middle	never	never	T3	N3	M0	IIIc	Yes	L1: left gastric node; L2: paracardial node; L3: pretracheal node	Deceased	8
ESCC009	Female	55	G2	Lower	never	never	T3	N3	M0	IIIc	Yes	L1: pretracheal node; L2: right recurrent laryngeal nerve node	Living	22
ESCC012	Male	65	G2	Middle	moderate	never	T3	N1	M0	IIIa	Yes	L1: pretracheal node; L2: middle paraesophageal node; L3: right recurrent laryngeal nerve node	Deceased	2
ESCC013	Male	64	G2-3	Middle	never	never	T3	N1	M0	IIIa	Yes	L1: left gastric node	Living	14
ESCC019	Male	69	G2-3	Middle	heavy	heavy	T2	N0	M0	II b	Yes	L1: subcarinal node 1; L2: subcarinal node 2; L3: left gastric node	Deceased	9
ESCC020	Male	63	G3	Middle	heavy	moderate	T3	N3	M0	IIIc	Yes	L1: subcarinal node; L2: middle paraesophageal node; L3: left gastric node	Deceased	12
ESCC022	Female	65	G2-3	Middle	moderate	never	T3	N3	M0	IIIc	Yes	L1: subcarinal node; L2: left recurrent laryngeal nerve node; L3: pretracheal node	Deceased	9
ESCC023	Male	72	G3	Lower	never	never	T3	N2	M0	IIIb	Yes	L1: paracardial node 1; L2: paracardial node 3; L3: paracardial node 4	Living	16
ESCC030	Male	52	G3	Middle	never	never	T3	N1	M0	IIIa	Yes	L1: left gastric node	Living	14
ESCC035	Female	62	G2	Middle	never	never	T3	N2	M0	IIIb	Yes	L1: splenic node; L2: left gastric node; L3: common hepatic node	Deceased	11
ESCC036	Female	58	G2	Middle	never	never	T3	N2	M0	IIIb	Yes	L1: middle paraesophageal node; L2: right recurrent laryngeal nerve node; L3: lower paraesophageal node	Deceased	7

Table S4. Differentially expressed N-glycosite-containing peptides in N-glycoproteomics analysis normalized by proteomics analysis.

Protein accession	Position	v13-TSTA3/Con Ratio	Regulated Type	Amino acid	Gene name	Localization probability	PEP	Score	Modified sequence	Charge	Mass error [ppm]	MS/MS Count	Con	v13-TSTA3
O00592	360	0.123	Down	N	PODXL	1	2.73095E-18	161.95	QLVLN(1)LTGN(1)TLCAGGASDEK	3	-0.44236	6	1.603	0.197
O00754	930	0.292	Down	N	MAN2B1	1	1.09982E-07	137.89	N(1)LSAPVTLNLR	2	0.37984	4	1.441	0.421
O14773	443	0.253	Down	N	TPP1	1	1.89829E-18	165.16	FLSSSPHLPPSSYFN(1)ASGR	3	2.066	20	1.483	0.375
O15031	733	0.364	Down	N	PLXNB2	1	3.75062E-10	142.32	LSHDAN(1)ETLPLHLYVK	2	0.69519	6	1.384	0.503
O15031	759	0.07	Down	N	PLXNB2	1	6.0231E-11	147.91	LHVTLYN(1)CSFGR	3	-0.15837	3	1.682	0.118
O15031	1099	0.211	Down	N	PLXNB2	1	1.23402E-11	120.71	TEAGAFEYVPDPTFEN(1)FTGGVK	2	-0.4368	2	1.524	0.322
O15118	158	0.084	Down	N	NPC1	0.998617	1.3891E-12	132.25	ELQYYVGQSFAN(0.001)AMYN(0.999)ACR	3	0.33449	1	1.606	0.135
O15230	243	0.418	Down	N	LAMA5	1	1.47969E-11	156.14	PGAMN(1)FSYSPLLR	2	-0.50979	1	1.369	0.572
O15230	95	0.481	Down	N	LAMA5	1	3.95642E-05	85.469	LVGGPVAGGDPN(1)QTIR	3	0.33467	3	1.317	0.633
O15230	2196	2.071	Up	N	LAMA5	1	3.19669E-11	158.79	GIN(1)ASSMAWAR	2	-0.33316	2	0.669	1.384
O75054	101	0.388	Down	N	IGSF3	1	1.8197E-09	132.81	VQGN(1)STLLHITDLQAR	3	0.84266	5	1.480	0.575
O75054	700	0.283	Down	N	IGSF3	1	2.25769E-10	142.91	TLTLVENKPIQLN(1)CSVK	3	-0.29664	5	1.613	0.457
O75054	842	0.498	Down	N	IGSF3	1	1.78191E-08	143.96	QVQLECVVLN(1)R	2	-0.51882	3	1.363	0.679
O75477	106	2.864	Up	N	ERLIN1	1	0.000146943	99.442	N(1)YTADYDK	2	0.20349	2	0.557	1.596
O75976	955	0.213	Down	N	CPD	0.999967	1.26534E-15	133.92	GLVMNYPHITN(1)LTNLGQSTEYR	3	-0.61666	4	1.790	0.381
O75976	978	0.257	Down	N	CPD	0.998291	1.50187E-08	107.15	HIWSLEISN(0.002)KPN(0.998)VSEPEEPK	4	0.19298	3	1.714	0.441
O96005	295	0.415	Down	N	CLPTM1	1	4.52669E-12	151.22	DYYPIN(1)ESLASLPLR	3	-0.1112	5	1.493	0.620
P02545	281	0.118	Down	N	LMNA	0.93193	4.50184E-07	117.3	N(0.932)SN(0.068)LVGAAHEELQQSR	3	0.43272	4	1.557	0.184
P02786	251	0.02	Down	N	TFRC	1	4.47245E-20	169.94	KDFEDLYTPVN(1)GSIVIVR	3	0.64975	8	1.702	0.034
P05026	158	0.328	Down	N	ATP1B1	1	3.29214E-10	132.64	LEWLGN(1)CSGLNDETYGYK	2	-0.41739	6	1.369	0.450
P05186	230	2.398	Up	N	ALPL	1	3.14812E-42	242.8	N(1)KTDVEYESDEK	3	0.15716	4	0.612	1.467
P05187	144	2.366	Up	N	ALPP	1	5.6009E-17	176.09	FNQCN(1)TTR	2	-0.2859	5	0.674	1.595
P05362	183	2.041	Up	N	ICAM1	1	2.1056E-07	127.37	DHHGAN(1)FSCR	3	0.40728	3	0.682	1.391
P05556	481	3.177	Up	N	ITGB1	1	1.44227E-11	147.26	CHEGN(1)GTFECGACR	2	-0.76998	4	0.497	1.578
P05556	520	2.616	Up	N	ITGB1	0.999999	8.31467E-16	136.96	KEN(1)SSEICSNNGECVCGQCVCR	3	-1.3572	4	0.571	1.493
P06280	139	0.049	Down	N	GLA	1	0.000757736	85.355	LGIYADVGN(1)K	2	-1.6447	3	1.702	0.084
P07602	215	0.318	Down	N	PSAP	1	2.52938E-32	207.07	TN(1)STFVQALVEHVKEECDR	2	0.072779	11	1.443	0.459
P07602	332	2.154	Up	N	PSAP	1	2.5228E-28	205.88	LIDN(1)N(1)KTEK	2	0.43279	23	0.658	1.418
P07602	80	0.068	Down	N	PSAP	1	3.22466E-09	129.38	DVVTAAGDMLKDN(1)ATEEEILVYLEK	2	0.61484	8	1.722	0.118
P07602	331	0.301	Down	N	PSAP	1	2.20879E-09	129.88	LIDN(1)N(1)KTEK	2	0.43279	2	1.459	0.439
P07910	230	0.451	Down	N	HNRNPC	1	0.000181425	89.624	N(1)DKSEEEQSSSSVK	3	-0.0058328	1	1.331	0.601
P08195	365	0.44	Down	N	SLC3A2	1	1.4141E-23	179.58	DIENLKDASSFLAEWQN(1)ITK	3	0.62498	14	1.372	0.603
P08236	272	3.266	Up	N	GUSB	1	6.95246E-17	169.89	VVAN(1)GTGTQGQLK	2	-0.1334	3	0.500	1.635
P10253	140	0.435	Down	N	GAA	1	1.10756E-11	121.03	LEN(1)LSSSEMGYTATLTR	3	-0.44035	4	1.251	0.544
P11047	1223	0.186	Down	N	LAMC1	1	5.40275E-15	165.45	TLAGEN(1)QTAFEIEELNR	3	-0.033576	3	1.937	0.359
P11117	331	0.23	Down	N	ACP2	1	5.25427E-07	101.05	N(1)ESDKAPWPLSLPGCPHR	3	-0.050025	11	1.496	0.345
P11142	584	0.45	Down	N	HSPA8	1	7.02332E-12	127.71	N(1)QTAEKEEFEHQQK	3	-0.2094	3	1.470	0.661
P11166	45	0.428	Down	N	SLC2A1	1	7.88207E-08	138.24	VIEEFYN(1)QTWVHR	3	0.2203	2	1.251	0.536
P11279	84	0.328	Down	N	LAMP1	1	5.27139E-32	206.63	SSCGKEN(1)TSDPSLVIAFGR	3	-0.70058	13	1.604	0.526
P11279	103	0.347	Down	N	LAMP1	1	2.107E-16	167.76	GHTLTLN(1)FTR	3	-0.062803	9	1.578	0.547
P11279	76	0.349	Down	N	LAMP1	1	1.25019E-13	145.69	SGPKN(1)MTFDLPSDATVVLN(1)R	2	-0.23382	14	1.575	0.550
P11279	62	0.233	Down	N	LAMP1	1	1.25019E-13	145.69	SGPKN(1)MTFDLPSDATVVLN(1)R	2	-0.23382	16	1.754	0.409
P11717	1656	0.399	Down	N	IGF2R	1	5.1346E-12	160.18	N(1)GSSIVDLSPLIHR	3	0.41191	4	1.412	0.563
P11717	112	0.135	Down	N	IGF2R	1	9.39128E-06	78.917	SLLEFN(1)TTVSCDQQGTNHR	3	-0.74441	2	1.722	0.233
P12270	2036	0.294	Down	N	TPR	0.804719	1.05701E-08	94.389	AADSQN(0.805)SGEGN(0.195)TGAAESSFSQEVSR	2	0.95566	1	1.413	0.415
P13473	275	2.673	Up	N	LAMP2	1	0.0045214	77.923	LN(1)SSTIK	2	0.20415	2	0.579	1.547
P13473	257	2.049	Up	N	LAMP2	0.999979	6.18677E-33	205.18	VASVININPN(1)TTHSTGSCR	2	-0.24003	9	0.687	1.407
P13667	328	0.49	Down	N	PDIA4	0.901577	0.00748758	46.838	GESDPAYQQYQDAAN(0.902)N(0.098)LR	3	3.3578	1	1.318	0.647
P13987	43	0.418	Down	N	CD59	1	1.19417E-06	107.98	TAVN(1)CSSDFDACLITK	3	-0.43287	20	1.296	0.541
P14625	217	0.184	Down	N	HSP90B1	0.87188	8.70943E-30	173.43	HN(0.128)N(0.872)DTQHIWESDSNEFSVIADPR	4	0.0045156	27	1.613	0.296
P14625	481	2.86	Up	N	HSP90B1	1	1.14529E-21	161.87	IADDKYN(1)DTFWK	2	0.80052	3	0.536	1.533
P14625	62	0.118	Down	N	HSP90B1	1	4.78353E-22	148.4	TDDEVVQREEEAIQLDGLN(1)ASQIR	3	0.64275	11	1.697	0.200
P14866	278	0.371	Down	N	HNRNPL	0.999962	1.45217E-14	106.12	N(1)DQDTWDYTNPNLSGQGDPGSNPNKR	3	-0.13145	4	1.371	0.509
P15144	128	2.363	Up	N	ANPEP	1	3.13221E-16	172.21	KLN(1)YTLSQGHR	2	2.8801	5	0.668	1.578
P15144	234	0.338	Down	N	ANPEP	1	0.0053736	60.255	AEFN(1)ITLIHPK	2	0.10904	2	1.319	0.445
P15151	120	0.109	Down	N	PVR	1	3.47382E-06	92.671	VEDEGN(1)YTCLFVTFPQGSR	3	-0.30759	1	1.562	0.171
P15328	69	4.104	Up	N	FOLR1	1	2.31655E-42	222.13	N(1)ACCSTN(1)TSQEAHKDVSYLYR	3	-0.46246	19	0.494	2.029
P15328	161	3.889	Up	N	FOLR1	1	2.09501E-07	125.68	GWN(1)WTSGFN(1)K	2	-0.34529	11	0.512	1.992
P15328	63	4.294	Up	N	FOLR1	1	2.24385E-18	159.75	N(1)ACCSTN(1)TSQEAHKDVSYLYR	3	-0.46246	6	0.479	2.059
P15529	83	2.291	Up	N	CD46	1	3.08978E-15	151.99	N(1)HTWLPVSDDACYR	2	0.25927	11	0.635	1.456
P15586	111	2.919	Up	N	GNS	0.996539	1.64538E-05	85.496	YPHNHHVVN(0.997)N(0.004)TLEGN(1)CSSK	3	0.50145	4	0.561	1.637
P15586	117	2.919	Up	N	GNS	0.999724	1.64538E-05	85.496	YPHNHHVVN(0.997)N(0.004)TLEGN(1)CSSK	3	0.50145	4	0.561	1.637
P16070	57	2.538	Up	N	CD44	1	1.16331E-07	127.3	AFN(1)STLPTMAQMEK	3	-0.15743	20	0.638	1.619
P16278	464	0.173	Down	N	GLB1	1	3.54903E-05	105.46	NNVITLN(1)ITGK	2	-0.38545	4	1.564	0.271
P17301	105	0.135	Down	N	ITGA2	1	4.90322E-05	81.656	LNLQTSTSIPN(1)VTEMK	2	-0.63026	3	1.802	0.243
P18084	347	0.008	Down	N	ITGB5	1	7.12657E-09	116.6	N(1)FTALIPGTTVEILDGDSK	3	0.25637	2	1.801	0.014
P21796	37	0.263	Down	N	VDAC1	0.999987	0.000201411	61.096	SEN(1)GLEFTSSGSANTETTK	3	-0.044522	2	1.532	0.402
P22626	326	0.301	Down	N	HNRNPA2B1	0.999399	3.99318E-08	81.974	N(0.999)MGGPYGGGN(0.001)YGPGGSGGSGGYGGR	2	-0.74139	5	1.434	0.432
P26006	935	0.306	Down	N	ITGA3	1	2.03088E-12	161.21	VWN(1)STFIEDYRDFDR	3	-0.078609	3	1.464	0.448
P26006	265	0.487	Down	N	ITGA3	1	4.99365E-08	140.49	N(1)ITIVTGAPR	2	-0.50561	4	1.305	0.636
P26006	573	0.125	Down	N	ITGA3	1	6.03994E-08	127.32	LRPIIISMN(1)YSLPLR	3	-0.82662	2	1.665	0.208
P28799	265	2.825	Up	N	GRN	1	2.35399E-05	113.69	EN(1)ATTDLLTK	2	0.53193	3	0.532	1.504
P32004	479	0.255	Down	N	L1CAM	1	2.56325E-07	126.19	FFPYAN(1)GTLGIR	2	0.32921	6	1.584	0.404
P32004	726	0.427	Down	N	L1CAM	0.999547	5.81281E-07	113.47	GEGN(1)ETTNMVITWKPLR	2	1.5101	4	1.396	0.596
P35613	268	2.953	Up	N	BSG	1	5.02566E-38	233.31	ITDSEDKALMN(1)GSESR	3	-0.3067	14	0.535	1.579
P42892	539	0.387	Down	N	ECE1	1	9.70385E-11	164.89	FFN(1)FSWR	2	0.033301	2	1.284	0.497

P48960	371	2.011	Up	N	CD97	1	7.25364E-16	170.79	GDKN(1)VTMGQSSAR	2	-0.89854	4	0.638	1.284
P48960	38	2.371	Up	N	CD97	1	2.41637E-12	157.86	WCPQN(1)SSCVN(1)ATACR	2	-0.78464	3	0.565	1.341
P48960	453	0.402	Down	N	CD97	0.990548	5.15026E-14	157.37	RLSAVNSIFLSHN(0.991)N(0.009)TK	3	-0.097874	11	1.504	0.605
P49321	707	0.291	Down	N	NASP	1	4.95618E-05	72.568	KPTDGASSSN(1)CVTDISHLVR	3	-0.68868	2	1.427	0.415
P50895	439	0.109	Down	N	BCAM	1	1.22566E-05	100.68	TQN(1)FTLLVQGSPELK	3	-0.19129	3	1.882	0.205
P50897	197	0.249	Down	N	PPT1	1	6.51909E-25	208.68	N(1)HSIFLADINQER	2	-0.092547	14	1.464	0.365
P50897	212	2.037	Up	N	PPT1	1	0.000660787	90.709	GIN(1)ESYKK	2	0.28273	4	0.696	1.417
P54289	824	0.047	Down	N	CACNA2D1	1	2.67637E-07	125.36	IDVNSWIEN(1)FTK	2	0.11491	2	1.674	0.079
P54709	124	0.12	Down	N	ATP1B3	1	8.24368E-18	182.72	N(1)LTVCPDGALFEQK	3	-0.10257	13	1.854	0.223
P62937	102	2.522	Up	N	PPIA	1	3.21013E-15	117.61	HTGPGILSMAN(1)AGPN(1)TN(1)GSQFFICTAK	4	-1.9546	3	0.550	1.387
P98160	3836	0.104	Down	N	HSPG2	1	2.87298E-08	80.082	IQGEEIVFHDLN(1)LTAHGISHCPTCR	4	0.34344	2	1.438	0.150
Q01082	2130	0.233	Down	N	SPTBN1	1	0.000166055	71.909	GEQVSQN(1)GLPAEQGSPR	2	-0.85548	1	1.563	0.364
Q02388	786	0.492	Down	N	COL7A1	1	8.01199E-12	164.48	LQILN(1)ASSDVLR	2	0.19662	2	1.243	0.612
Q02809	197	0.298	Down	N	PLOD1	1	1.46304E-16	174.41	EQIN(1)ITLDHR	3	0.26242	5	1.580	0.471
Q02809	538	2.46	Up	N	PLOD1	1	4.84053E-05	106.6	YIHQN(1)YTK	2	0.022728	3	0.567	1.395
Q08722	111	2.361	Up	N	CD47	1	9.11933E-33	202.07	SDAVSHTGN(1)YTCEVTELTR	3	-0.97374	4	0.648	1.531
Q10472	552	0.338	Down	N	GALNT1	1	4.29081E-05	108.46	N(1)VTLPEIF	2	0.042629	2	1.412	0.477
Q11206	310	3.03	Up	N	ST3GAL4	1	1.46228E-20	182.49	SMAGSGHN(1)VSQEALAIKR	3	0.48266	6	0.489	1.482
Q13421	523	0.154	Down	N	MSLN	1	3.26726E-09	123.02	ALSQQN(1)VSMDLATFMK	2	-2.9714	4	1.882	0.290
Q13510	286	0.015	Down	N	ASAH1	1	1.79925E-15	129.2	ILAPAYFILGGN(1)QSGEGCVITR	3	0.43748	5	2.116	0.032
Q13586	171	0.324	Down	N	STIM1	1	0.000015171	111.12	LAVTN(1)TTMTGTVLK	2	0.13711	3	1.437	0.465
Q13740	265	2.257	Up	N	ALCAM	1	6.57967E-05	119.88	NAIKEGDN(1)ITLK	2	0.22977	6	0.655	1.479
Q13740	480	0.123	Down	N	ALCAM	1	6.87138E-14	142.97	IIISPEEN(1)VTLTCTAENQLER	3	0.56205	6	1.580	0.195
Q13740	361	2.093	Up	N	ALCAM	1	4.84053E-05	106.6	N(1)ATVVWMK	2	-0.41661	6	0.686	1.436
Q13813	678	0.292	Down	N	SPTAN1	1	1.75618E-05	116.05	EAN(1)QQQQFNR	2	-0.67499	1	1.502	0.439
Q14108	105	0.255	Down	N	SCARB2	0.999925	0.000060684	75.564	ANIQFGDN(1)GTTISAVSNK	3	0.15506	3	1.489	0.379
Q15043	77	2.262	Up	N	SLC39A14	1	2.62992E-16	174.24	GN(1)VTQHVQGHR	2	-0.097299	4	0.594	1.343
Q15165	254	3.032	Up	N	PON2	0.99999	2.70552E-23	190.53	HTNMN(1)LTQLK	3	-0.31637	12	0.531	1.608
Q15293	53	0.149	Down	N	RCN1	1	5.82481E-27	149.16	VVRPDSELGERPPEDN(1)QSFQYDHEAFLGK	3	0.45239	16	1.863	0.278
Q15904	170	0.441	Down	N	ATP6AP1	1	1.40385E-07	134.75	LN(1)ASLPALLLIR	2	0.254	2	1.485	0.655
Q32P28	540	0.367	Down	N	P3H1	1	4.21358E-08	128.99	VPLQSAHLYYN(1)VTEK	2	0.72151	6	1.448	0.532
Q5ZPR3	104	0.06	Down	N	CD276	1	1.1101E-10	144.29	TALFPDLLAQGN(1)ASLR	3	0.41337	4	1.462	0.088
Q5ZPR3	91	4.095	Up	N	CD276	1	8.29377E-18	157.83	QLVHSFAEGQDQGSAYAN(1)R	3	-0.93915	1	0.490	2.007
Q6PIU2	287	0.108	Down	N	NCEH1	1	3.17668E-05	105.99	LN(1)WTSLLPASFTK	2	-0.21201	5	1.516	0.164
Q6YHK3	68	0.089	Down	N	CD109	1	1.51986E-10	139.88	TASN(1)LTVSVLEAEGVFEK	3	-0.28781	1	1.596	0.143
Q6ZRP7	266	0.061	Down	N	QSOX2	0.999954	1.20344E-26	147.11	LGVSSVPSCYLIYPN(1)GSHGLINVVKPLR	4	-0.32103	1	1.688	0.103
Q70UQ0	328	0.182	Down	N	IKBIP	1	1.81864E-07	131.69	ISN(1)LTIVQAEIK	2	0.15848	2	1.491	0.271
Q70UQ0	144	0.187	Down	N	IKBIP	0.963958	1.34237E-16	167.29	QDILN(0.964)N(0.036)SLTTLSQDITK	2	-0.90109	3	1.486	0.278
Q7Z4H8	251	0.048	Down	N	KDELC2	1	1.22219E-10	122.53	KVN(1)GTPSPIPIISWCGSLDSR	3	0.57242	1	1.618	0.077
Q7Z4H8	419	2.15	Up	N	KDELC2	1	0.000880752	88.177	N(1)LSDLLEK	2	-0.21198	2	0.684	1.471
Q8IV08	387	4.086	Up	N	PLD3	1	3.85681E-11	157.03	DN(1)HTHSDIQVK	2	-0.34154	3	0.451	1.844
Q8NBJ4	109	0.342	Down	N	GOLM1	0.997656	1.74349E-16	174.57	AVLVN(0.002)N(0.998)ITTGER	2	-0.2653	5	1.847	0.631
Q8NBJ5	96	0.432	Down	N	COLGALT1	0.999597	4.37779E-24	167.99	TALWVATDHNMDN(1)TSTVLR	3	-0.52293	5	1.342	0.580
Q8NC56	311	0.468	Down	N	LEMD2	1	5.33327E-06	77.959	CIPVMEAQEYIAN(1)VTSSSSAK	2	2.6003	2	1.278	0.598
Q8NHP8	465	2.376	Up	N	PLBD2	1	4.14824E-12	164.48	RN(1)QSLVQDMDSMVR	2	0.34502	9	0.646	1.536
Q8TEM1	405	0.02	Down	N	NUP210	1	5.18703E-22	152	IETVLPAEFFEVLSSSQN(1)GSYHR	3	0.068834	5	1.706	0.034
Q8TEM1	44	0.083	Down	N	NUP210	1	5.9392E-17	172.76	VN(1)FTLEASEGCYR	2	0.048265	2	1.633	0.135
Q92542	387	0.054	Down	N	NCSTN	1	2.03264E-13	142.88	TSLELWMHTDPVSQKN(1)ESVR	3	-1.317	5	1.646	0.088
Q92542	530	2.595	Up	N	NCSTN	1	2.21533E-32	223.78	AN(1)N(1)SWFQSILR	2	1.0374	6	0.602	1.562
Q93050	488	0.165	Down	N	ATP6V0A1	1	6.78601E-06	119.39	PMFTYN(1)WTEETLR	2	-0.52928	2	1.607	0.265
Q969N2	327	0.144	Down	N	PIGT	1	5.67517E-14	147.64	KTYAIYDLLDTAMIN(1)N(1)SR	3	-0.68107	9	1.828	0.263
Q96AY3	294	2.082	Up	N	FKBP10	1	6.86634E-69	274.43	YHYN(1)GSLMDGTLFDSSYSR	2	-1.1303	6	0.644	1.340
Q96AY3	310	0.385	Down	N	FKBP10	0.999805	8.2321E-28	164.35	N(1)HTYNTYIGQGYIIPGMDQGLQGACMGER	3	-0.75647	5	1.459	0.562
Q96AY3	393	2.176	Up	N	FKBP10	1	3.32624E-17	186.77	TLSRPSETCN(1)ETTK	2	-0.11888	7	0.624	1.359
Q96AY3	70	2.635	Up	N	FKBP10	1	1.79473E-16	151.79	YHYN(1)GTFEDGKK	2	-0.092825	10	0.545	1.435
Q96AY3	407	0.231	Down	N	FKBP10	1	5.07926E-07	71.903	YHYN(1)CSLLDGTQLFTSHDYGAPQEATLGANK	3	-0.34616	1	1.648	0.381
Q96D15	140	0.449	Down	N	RCN3	1	1.10274E-11	119.34	N(1)ATYGHYAPGEEFHDVEDAETYKK	4	-0.085379	10	1.229	0.552
Q96HE7	280	2.496	Up	N	ERO1A	1	1.58908E-23	201.84	WGHN(1)ITEFQQR	2	-1.0022	7	0.622	1.554
Q99538	272	2.692	Up	N	LGMN	1	6.27502E-25	195.94	SHTN(1)TSHVMQYGN(1)K	3	0.70954	6	0.576	1.550
Q99538	263	2.692	Up	N	LGMN	1	6.27502E-25	195.94	SHTN(1)TSHVMQYGN(1)K	3	0.70954	6	0.576	1.550
Q9H1E3	53	0.275	Down	N	NUCKS1	1	2.71433E-07	135.61	N(1)SQEDSEDSEDKDVK	2	-0.47971	1	1.384	0.380
Q9HAT2	401	0.252	Down	N	SIAE	1	2.68024E-14	145.91	N(1)LTFEGPLPEK	2	0.36313	7	1.277	0.321
Q9HDC9	196	0.156	Down	N	APMAP	1	2.48277E-25	194.48	N(1)MSFVNDLTVTQDGRK	3	0.60492	14	1.540	0.241
Q9NWM8	176	0.499	Down	N	FKBP14	1	1.21118E-52	191.22	HGAVVN(1)ESHHDALVEDIFDKEDEDKDGFISAR	5	-0.50287	4	1.285	0.641
Q9NX62	259	0.481	Down	N	IMPAD1	1	4.216E-11	115.42	QVALQTFGN(1)QTTIIPAGGAGYK	2	0.58798	4	1.435	0.690
Q9P2B2	286	2.39	Up	N	PTGFRN	1	0.00522913	80.787	N(1)VSVAEGK	1	-0.1283	2	0.634	1.515
Q9P2B2	618	2.281	Up	N	PTGFRN	1	7.72974E-12	157.96	LEN(1)WTDASR	2	-0.17352	2	0.653	1.489
Q9P2B2	300	0.208	Down	N	PTGFRN	1	5.56533E-11	147.58	ELDLTCN(1)ITTDR	2	2.3595	3	1.490	0.310
Q9UBV2	272	0.426	Down	N	SEL1L	1	1.05989E-24	173.56	GQTALGFLYASGLGVN(1)SSQAK	3	-0.046738	4	1.373	0.585
Q9UHL4	315	0.311	Down	N	DPP7	1	5.50944E-18	157.47	ALAGLVYN(1)ASGSEHCYDIYR	2	1.7776	7	1.440	0.448
Q9UHN6	525	0.316	Down	N	TMEM2	1	0.00118622	71.879	N(1)FTSVHLSYVELK	3	1.3486	2	1.605	0.507
Q9UIQ6	834	0.339	Down	N	LNPEP	0.989704	2.8671E-09	114.46	SALLEFACTHN(0.01)LGN(0.99)CSTTAMK	3	-0.27185	2	1.454	0.494
Q9UIQ6	184	0.065	Down	N	LNPEP	1	0.000131683	81.448	YELSLHPN(1)LTSMTFR	3	-0.46804	1	1.792	0.117
Q9UN86	59	0.204	Down	N	G3BP2	0.999999	3.48497E-06	61.538	NSSYVHGGVDASGKPQEAVYGQN(1)DIHHK	4	0.018783	2	1.814	0.371
Q9UP95	312	0.149	Down	N	SLC12A4	1	6.91751E-07	115.06	SIFDPPVFPVCMLGN(1)R	3	-0.15906	6	1.562	0.233
Q9Y4L1	830	0.143	Down	N	HYOU1	0.999999	3.49629E-20	179.66	LSALDNLLN(1)HSSMFLK	2	-0.044032	10	1.627	0.233
Q9Y4L1	869	2.405	Up	N	HYOU1	1	1.45159E-07	128.36	N(1)ATLAEQAK	2	0.11519	2	0.613	1.473
Q9Y639	284	3.053	Up	N	NPTN	1	6.53874E-05	88.19	KKENGMPMDIVN(1)TSGR	3	0.17214	5	0.537	1.639
Q9Y6M0	167	0.148	Down	N	PRSS21	1	1.65203E-09	126.07	HIQPICLQASTFEFEN(1)R	2	0.14945	5	2.113	0.314

Table S5. Primers and siRNA used in this study.

Primer Name	Forward Primer Sequence (5' to 3')	Reverse Primer Sequence (5' to 3')	Used for
TSTA3-primer 1	5'-GCGGATTCTAGTGACAGGGG	GATGGATG ACGTGTGTG GGT	RT-PCR for TSTA3 expression analysis
GAPDH-primer 2	CGGAGTCAACGGATTTGGTCGTAT	AGCCTTC TCCATGGTGGTGAAGAC	RT-PCR for GAPDH expression analysis
TSTA3-sh1	CDS:CCGGTCCGGAATATCAAATACAATTCTCGAGAAT TGTATTTGAT ATTCCGG A TTTTTG		TSTA3 Knockdown
TSTA3-sh2	UTR:CCGGAGCTGGAAGACAGGATCAGGTCTCGAGACCTGATCCTGTCT TCCAGCTTTTTG		TSTA3 Knockdown
LAMP2-siRNA1	CTGGGATGTTCTTGTACAA		LAMP2 Knockdown
LAMP2-siRNA2	CCACTTGCCTTTATGCAAA		LAMP2 Knockdown
LAMP2-siRNA3	GCGGTCTTATGCATTGGAA		LAMP2 Knockdown
ERBB2-siRNA1	GGAGACCCGCTGAACAATA		ERBB2 Knockdown
ERBB2-siRNA2	CAGACACGTTTGAGTCCAT		ERBB2 Knockdown
ERBB2-siRNA3	GGAAGGACATCTTCCACAA		ERBB2 Knockdown
